# Nanoparticle-Mediated Nose-to-Brain Delivery for Ischemic Stroke Therapy: Preclinical Insights

**DOI:** 10.3390/pharmaceutics17111447

**Published:** 2025-11-09

**Authors:** Joonhyuck Park, Tae-Ryong Riew

**Affiliations:** 1 Department of Medical Life Sciences, College of Medicine, The Catholic University of Korea, Seoul 06591, Republic of Korea; joonpark@catholic.ac.kr; 2 Department of Medical Sciences, College of Medicine, Graduate School, The Catholic University of Korea, Seoul 06591, Republic of Korea; 3 Department of Anatomy, Catholic Neuroscience Institute, College of Medicine, The Catholic University of Korea, Seoul 06591, Republic of Korea

**Keywords:** ischemic stroke, intranasal drug delivery, nose-to-brain transport, blood-brain barrier, nanomaterial

## Abstract

Ischemic stroke remains a major cause of mortality and long-term disability, yet current therapeutic strategies are largely limited to reperfusion approaches such as intravenous thrombolysis and thrombectomy, which are constrained by narrow treatment windows and the risk of complications. Moreover, the blood–brain barrier (BBB) severely restricts drug penetration into the injured brain, limiting the translation of promising neuroprotective agents into clinical success. Intranasal (IN) delivery has emerged as a compelling alternative route that bypasses the BBB and enables rapid access to the central nervous system through olfactory, trigeminal, and perivascular pathways. This narrative review highlights recent advances in preclinical research on IN therapeutics for ischemic stroke, ranging from small molecules and biologics to nucleic acids and cell-based therapies. Particular emphasis is placed on the application of nanotechnology, including extracellular vesicles, liposomes, and inorganic nanoparticles, which enhance drug stability, targeting, and bioavailability. Studies demonstrate that IN delivery of growth factors, cytokines, and engineered stem cells can promote neurogenesis, angiogenesis, white matter repair, and functional recovery, while nanocarriers further expand the therapeutic potential. Overall, intranasal delivery represents a promising and non-invasive strategy to overcome the limitations of conventional stroke therapies, offering new avenues for neuroprotection and regeneration that warrant further investigation toward clinical translation.

## 1. Introduction

Ischemic stroke affects more than 12 million people worldwide annually and remains one of the leading causes of death and long-term disability. Responsible for seven million deaths annually, it is a major global health burden, leaving the majority of over 80 million survivors with significant long-term disabilities [1,2,3]. Existing treatments are largely confined to reperfusion strategies, such as intravenous thrombolysis with tissue plasminogen activator (tPA) and mechanical thrombectomy. However, these strategies are restricted by a narrow therapeutic time window and associated risks of hemorrhagic complications [1,4]. Consequently, the majority of patients are left without effective interventions, underscoring the urgent need for novel therapeutic approaches that are both more effective and widely applicable. These approaches should not only protect neural tissue during the acute phase but also promote repair and functional recovery in the subacute and chronic stages of stroke [5,6]. Compounding this challenge, the blood–brain barrier (BBB) severely restricts drug penetration into the injured brain. This prevents many promising neuroprotective agents from achieving therapeutic efficacy and hindering the translation of preclinical success into clinical outcomes [7,8].

To overcome these limitations, intranasal (IN) delivery has emerged as a compelling non-invasive strategy that bypasses the BBB and provides direct access to the central nervous system (CNS) [9,10,11,12]. The unique anatomical and physiological characteristics of the nasal cavity—including its close proximity to the brain, rich vascularization, and direct neuronal connections via the olfactory and trigeminal nerves—allow drugs to reach cerebral tissues within minutes [9,12,13,14]. To leverage this route, nanoparticle-based therapeutics (NBTs) have emerged as a particularly promising strategy. Nanocarriers offer numerous beneficial points for nose-to-brain delivery; they can protect sensitive therapeutic cargo, such as biologics and nucleic acids, from rapid enzymatic degradation within the nasal mucosa [15,16]. Furthermore, they can be engineered to overcome challenges like rapid mucociliary clearance by enhancing bioavailability, prolonging half-life, and improving targeting efficiency, ultimately maximizing drug concentrations in the brain [17,18]. Building on these advantages, recent preclinical studies have expanded the scope of IN therapeutics for ischemic stroke, spanning small molecules, peptides, proteins, nucleic acids, and even cell-based therapies, often enhanced through the use of advanced nanocarrier systems [7,19,20,21]. Collectively, these advances highlight the promise of intranasal delivery as both a neuroprotective and regenerative therapeutic modality, while also emphasizing the need for further investigation into its mechanisms, efficacy, and translational feasibility.

**Figure 1:**
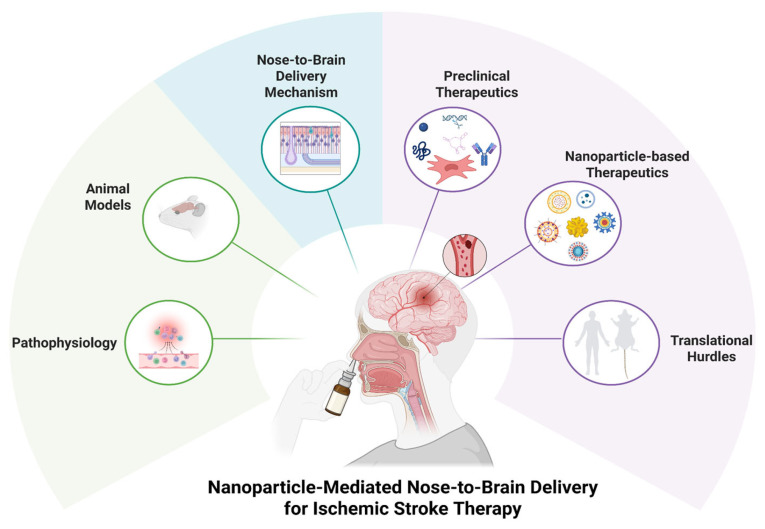
Illustration of nanoparticle-mediated nose-to-brain delivery for ischemic stroke therapy.

In this review, we first outline the pathophysiology of ischemic stroke as the basis for identifying novel therapeutic targets, then provide an overview of intranasal anatomy and nose-to-brain delivery mechanisms, and finally summarize emerging preclinical therapeutics and nanocarrier systems (Figure 1). Through this, we aim to explore the potential of intranasal delivery as a novel strategy for the development of effective treatments for ischemic stroke, a disease area where clear therapeutic breakthroughs remain elusive.

## 2. Pathophysiology of Ischemic Stroke

An acute ischemic stroke, caused by a sudden blockage of blood flow to the brain, triggers a complex series of events that can be broken down into three phases: acute cell death, subacute inflammation, and long-term recovery and regeneration.

### 2.1. Acute Cell Death

The immediate aftermath of an ischemic stroke is characterized by the rapid death of brain cells through two primary mechanisms: oxidative stress and excitotoxicity.

#### 2.1.1. Oxidative Stress

When blood flow is cut off, cells are starved of oxygen and glucose. This leads to a breakdown in cellular function, but the real damage often occurs upon reperfusion, or the return of blood flow. Reperfusion causes a massive surge of reactive oxygen species (ROS) [22]. While a healthy cell’s mitochondria produce small amounts of ROS, the oxygen deprivation during ischemia disrupts this process. This leads to acidosis from lactic acid buildup, which further impairs antioxidant defenses. Reperfusion then triggers an explosion of ROS, mainly from mitochondrial respiratory chain complex I and Nicotinamide adenine dinucleotide phosphate (NADPH) oxidase (NOX), an enzyme whose inhibitor, casein kinase 2 (CK2), is downregulated [22,23]. This unchecked oxidative stress damages lipids, proteins, and DNA, leading to a breakdown of cellular integrity. A key event is the activation of the mitochondrial permeability transition pore (MPTP) [24]. High levels of intracellular calcium during ischemia cause a calcium overload in the mitochondria, leading to MPTP opening. This pore opening halts mitochondrial respiration and accelerates cell death. Oxidative stress can also directly or indirectly activate cell death pathways mediated by proteins like p53, c-Jun N-terminal kinase (JNK), p38 mitogen-activated protein kinase (MAPK), and Poly(ADP-ribose) polymerase 1 (PARP-1) [22,24].

#### 2.1.2. Excitotoxicity

This process, mediated by the neurotransmitter glutamate, is a central player in stroke pathology. The brain is rich in glutamate and its receptors. During ischemia, a massive, uncontrolled release of glutamate occurs. This over-stimulates N-methyl-D-aspartate (NMDA) receptors (NMDARs), leading to a flood of calcium ions into the neurons [25,26]. While synaptic NMDARs are typically pro-survival, the activation of extrasynaptic NMDARs by the excessive glutamate promotes cell death. In the ischemic core region, complete energy depletion prevents cells from maintaining ion balance, leading to cell death [5,25]. In the surrounding ischemic penumbra, glutamate release from stressed neurons amplifies and spreads the damage by causing ionic imbalances, oxidative stress, and metabolic dysfunction [27].

### 2.2. Subacute Neuroinflammation and Reactive Gliosis

Following the acute phase, neuroinflammation contributes to further damage and cell death. Cell death in the ischemic core releases pro-inflammatory damage-associated molecular patterns (DAMPs), initiating a sterile inflammation response [6,27]. This activates resident immune cells like microglia and recruits peripheral immune cells (neutrophils, monocytes) to the brain. Microglia are the first to respond in the central nervous system, phagocytizing of cellular debris and releasing cytokines and chemokines [2]. Neutrophils are among the first peripheral cells to enter the brain, contributing to blood–brain barrier (BBB) damage and tissue injury by releasing neutrophil extracellular traps (NETs) and inflammatory mediators. Monocyte-derived macrophages (MDMs) also infiltrate the brain early on and differentiate into pro-inflammatory types [6]. T and B lymphocytes enter the brain within days to weeks. Pro-inflammatory T-cell subtypes worsen stroke outcomes, and natural killer (NK) cells also contribute to the damage [6,27]. Activation of the complement system further fuels inflammation and tissue destruction [6]. In the subacute phase, reactive astrocytes form a glial scar around the lesion [28,29,30]. Late-stage microglia can shift to a pro-inflammatory phenotype, inducing an inflammatory response in astrocytes, which then secrete neurotoxic substances that hinder recovery. Chronic inflammation is neurotoxic and contributes to ongoing damage.

### 2.3. Recovery and Tissue Regeneration

Despite the initial damage, the brain possesses remarkable capacity for repair and regeneration, a process that is also heavily influenced by neuroinflammation and reactive gliosis. During the later stage of ischemic injury, microglia can shift to an anti-inflammatory phenotype, facilitating inflammation resolution by clearing dead cells and secreting nourishing factors such as IGF-1 [6,31]. Regulatory T cells (Tregs) are beneficial T-cell subtypes that promote neurogenesis and white matter repair through factors like amphiregulin and osteopontin [32,33]. B lymphocytes can also play a protective role by producing anti-inflammatory cytokines like IL-10 [6,34].

Moving beyond the inflammatory response, the glial scar, formed mainly by reactive astrocytes and other neuroglial cells, initially acts as a protective barrier, isolating the damaged tissue and compartmentalizing the immune response [28,35]. However, it can also inhibit neural regeneration due to the release of growth-inhibiting factors like chondroitin sulfate proteoglycans [36,37]. On the other hand, matrix metalloproteinases (MMPs) in the recovery phase can mediate angiogenesis, neurogenesis, and synaptic remodeling [36]. Reactive astrocytes can also acquire neural stem cell-like characteristics and can be reprogrammed into neurons [38,39].

A critical repair process is angiogenesis, which helps restore blood flow and partially repair the blood–brain barrier (BBB) [40]. Vascular endothelial growth factor (VEGF) is a key promoter of angiogenesis, but in the early phase, it can also increase BBB permeability and the risk of bleeding. Pericytes, cells that maintain BBB integrity, can differentiate into neural and vascular cells and play a crucial role in re-establishing tight junctions to restore the BBB [41]. Remyelination of axons is another crucial recovery process in degenerated white matter. Tregs and astrocytes can promote the formation of new oligodendrocytes via osteopontin and Brain-Derived Neurotrophic Factor (BDNF), aiding in white matter repair and functional recovery [32,36,42].

Finally, neuroplasticity is the brain’s ability to rewire and reorganize neural circuits in response to injury. This process is most active in the first few months after a stroke [40]. It involves the sprouting of new axons, the formation of new synapses, and the remodeling of existing connections. Axon growth inhibitory factors are downregulated, and pro-growth factors like growth differentiation factor 10 (GDF10) are upregulated [43]. In addition, synaptic and dendritic spines are initially lost but regenerate during the recovery phase in the peri-infarct area [44]. The overall recovery of function involves the expansion of dendritic branches, the formation of new connections, and the sprouting of axons from the undamaged side of the brain to support the damaged area.

## 3. Commonly Used Preclinical Models of Ischemic Stroke

Diverse animal models have been developed to elucidate the complex pathophysiology of ischemic stroke and to discover novel molecules with therapeutic potential. The primary objective of any preclinical stroke model is to faithfully replicate the cardinal features of the human condition. This includes the formation of a necrotic infarct core, where cellular energy failure leads to rapid and irreversible cell death, and a surrounding ischemic penumbra. This penumbral region, characterized by reduced cerebral blood flow and metabolic dysfunction, is functionally silent but structurally intact and, therefore, potentially salvageable [4,45,46]. It is the principal target for neuroprotective and neurorestorative interventions. Recognizing that no single model can encapsulate the full heterogeneity of clinical stroke, the selection of an appropriate model is a critical decision dictated by the specific scientific question—be it the efficacy of an acute neuroprotectant, the mechanisms of reperfusion injury, or the potential for long-term functional recovery.

### 3.1. Focal Ischemia Models

Focal ischemia models are the most clinically relevant and widely utilized category in stroke research, as they mimic the occlusion of a specific cerebral artery, which accounts for the majority of human strokes. Among these, the Middle Cerebral Artery Occlusion (MCAO) model stands as the “gold standard,” a status reflected by its predominant use in the preclinical studies summarized throughout this review. The MCAO model’s strength lies in its ability to generate a large, reproducible infarct affecting both the cortex and the underlying striatum, closely recapitulating the vascular territory of the human middle cerebral artery [8,46,47]. The most common method for inducing MCAO is the intraluminal filament technique [48]. This procedure involves the insertion of a silicone-coated monofilament through the external carotid artery. The filament is then advanced into the internal carotid artery to physically block the origin of the MCA, thereby interrupting blood flow to a significant portion of the cerebral hemisphere. This interruption of blood flow leads to the formation of lesions in the territory supplied by the middle cerebral artery. This territory includes deep subcortical structures (like the basal ganglia, internal capsule, and thalamus), and the overlying frontotemporal and parietal cortices [8,47].

The MCAO model is further refined into distinct variants that correspond to different clinical scenarios. The transient MCAO (tMCAO) model involves withdrawing the filament after a defined period of occlusion, typically ranging from 60 to 90 min, to allow for reperfusion [49,50]. This variant is of paramount importance because it simulates the clinical sequence of successful recanalization therapy, such as intravenous thrombolysis with tissue plasminogen activator (tPA) or mechanical thrombectomy. As discussed in the previous section, the restoration of blood flow, while essential, paradoxically triggers a secondary wave of injury driven by a massive surge in reactive oxygen species (ROS), inflammation, and blood–brain barrier disruption [22]. Consequently, the tMCAO model is an essential experimental paradigm for evaluating therapies designed to mitigate this ischemia–reperfusion injury. In contrast, the permanent MCAO (pMCAO) model, where the filament is left in place, simulates clinical situations where reperfusion is not achieved. This results in a more severe and consistently larger infarct, providing a platform to study the maximal extent of ischemic damage and the brain’s endogenous responses without the confounding variable of reperfusion injury. A third variant, the distal MCAO (dMCAO), involves a craniotomy to directly ligate or cauterize a distal branch of the MCA. dMCAO offers a more invasive but highly precise method for producing a purely cortical infarct [50,51].

An alternative and increasingly utilized method for inducing focal ischemia is the photothrombotic (PT) model. This technique operates on a different principle than mechanical vessel occlusion. It involves systemic administration of a photosensitive dye called Rose Bengal. Subsequent illumination of a targeted cortical region through the intact skull or a thinned-cranial window activates the dye [52,53]. This activation generates singlet oxygen from the dye, which then induces endothelial cell damage, platelet aggregation, thrombus formation, and the occlusion of microvessels within the illuminated area. The resulting infarct is highly circumscribed, reproducible in size and location, and largely confined to the cortex, with minimal primary damage to the underlying white matter and a very limited penumbra [47,52]. While the MCAO model is superior for assessing acute neuroprotective agents aimed at salvaging the penumbra, the PT model’s precision allows researchers to meticulously track long-term structural and functional changes in the peri-infarct cortex. The predictability of the lesion size and location facilitates studies on axonal sprouting, synaptic remodeling, and the reorganization of cortical maps over weeks to months [47,54]. Therefore, the selection of the PT model often signifies a shift in research focus from acute neuroprotection to subacute and chronic neuroregeneration.

### 3.2. Translational Considerations and Model Limitations

Despite their indispensability, it is crucial to acknowledge the inherent limitations of these preclinical models and the challenges they pose for clinical translation. The failure of many promising neuroprotective agents in clinical trials is partly attributed to the “translational chasm” between animal models and the human clinical condition. Preclinical stroke research is predominantly conducted in young, healthy, male rodents under highly controlled experimental conditions [8]. In contrast, the typical human stroke patient is elderly and presents with comorbidities, such as hypertension, diabetes, atherosclerosis, and hyperlipidemia, all of which profoundly alter the brain’s vulnerability to ischemia and its response to therapy [55]. For example, a study comparing spontaneously hypertensive rats (SHR) with normotensive rats (NTR) revealed marked differences in infarct patterns and severity [56]. Furthermore, the controlled nature of experimental stroke induction—with a fixed occlusion time and often immediate therapeutic intervention—does not reflect the clinical variability in stroke onset, severity, and time to treatment [8]. In addition, there are structural differences in the brain. The rodent brain contains less than 10% white matter, while the brains of humans, non-human primates (NHPs), and pigs contain over 60%. This disparity makes it challenging to replicate subcortical white matter strokes in rodents, which constitute approximately 25% of all human strokes [8]. Nevertheless, the strategic selection of models allows researchers to ask specific and relevant questions. The MCAO model remains the most appropriate choice for assessing acute neuroprotectants and therapies targeting the multifaceted injury cascades associated with reperfusion. The PT model, in turn, provides an unparalleled system. It is ideal for dissecting the molecular and cellular mechanisms of long-term cortical reorganization and repair. Despite their limitations, these rodent models provide the essential and standardized platforms upon which the proof-of-concept and efficacy of the novel intranasal therapeutics discussed in the subsequent sections are built and rigorously evaluated.

## 4. Conventional Delivery Pathways in Preclinical Stroke Therapy

Other than conventional clinical treatment options like thrombolytic therapy or mechanical thrombectomy, numerous preclinical studies have been performed to investigate the therapeutic potential of neuroprotective and neurorestorative agents. A critical challenge in translating these promising agents into clinical success is the development of effective delivery strategies that can navigate the formidable blood–brain barrier (BBB). Various administration routes have been explored in preclinical settings, each with a distinct profile of advantages and limitations.

### 4.1. Systemic Delivery Routes: IV and IA Administration

Intravenous (IV) administration is the most common and minimally invasive route for systemic drug delivery. It allows for widespread distribution throughout the body, but this becomes a major drawback for CNS disorders. The BBB severely restricts the passage of most therapeutics, especially large biologics like proteins, nucleic acids, and cell-based therapies, resulting in suboptimal drug concentrations in the brain [7,57]. This low bioavailability necessitates higher systemic doses, which in turn increases the risk of off-target side effects [58]. For instance, systemically delivered mesenchymal stem cells (MSCs) often suffer from poor homing to the brain and reduced viability [57].

Intra-arterial (IA) administration offers a more targeted approach by delivering drugs directly into the cerebral circulation, bypassing the first-pass metabolism and achieving a higher local concentration in the brain compared to IV [57]. Preclinical studies have shown that IA delivery of stem cells can lead to superior recovery in terms of infarct volume reduction compared to other routes [59]. However, this method is highly invasive, requiring arterial catheterization by a skilled interventionalist. It also carries significant risks, including arterial dissection, hemorrhage, and distal emboli [57]. Moreover, even with IA delivery, therapeutics must still cross the BBB at the capillary level to reach the brain parenchyma.

### 4.2. Direct CNS Delivery

To circumvent the BBB entirely, direct injection into the central nervous system has been investigated. Intracerebroventricular (ICV) or intrathecal (IT) administration involves injecting therapeutics into the cerebrospinal fluid (CSF), allowing for broad distribution throughout the CNS [7,57,58]. This route has proven effective for delivering cell-based therapeutics in preclinical models [40,60]. However, it remains a highly invasive procedure requiring surgery or a lumbar puncture, posing risks of infection and procedural complications. Intraparenchymal or intracerebral (IC) injection into the brain parenchyma is the most direct method, ensuring the highest possible concentration at a specific target site [40,57]. While it completely bypasses the BBB, it is also the most invasive approach, carrying a substantial risk of tissue damage, inflammation, and hemorrhage at the injection site [57]. Furthermore, drug distribution is severely limited by slow diffusion from the point of injection, making it unsuitable for treating the widespread and diffuse damage characteristic of an ischemic stroke [61].

## 5. Nose-to-Brain Delivery Pathways and Mechanisms

### 5.1. Anatomy of the Intranasal Space

The nasal cavity anatomy crucial for drug delivery is divided into three main regions: the vestibular region, the respiratory region, and the olfactory region. The vestibular region is situated at the nasal entrance and is covered by non-keratinized stratified squamous epithelium. It contains nasal hairs, primarily filtering inhaled particles [12,62]. However, its small surface area suggests it contributes minimally to drug absorption [11]. The respiratory region is the most expansive area within the nasal cavity, constituting up to 90% of the human nasal cavity. It is lined by ciliated pseudostratified columnar epithelium with microvilli [11,12]. This region is rich in blood vessels, contains mucus-secreting goblet cells, and is innervated by the facial nerve and trigeminal nerve [62,63]. The olfactory region is located in the superior aspect of the nasal cavity. It covers the underside of the cribriform plate of the ethmoidal bone and is covered by pseudostratified columnar epithelium. In humans, this region accounts for about 10% of the total nasal surface area, while 50% in rodents [12,64]. The olfactory epithelium consists of Olfactory Sensory Neurons (OSNs), basal cells (acting as progenitor cells), and supporting cells [12,64].

Key structures within the nasal cavity that facilitate drug transfer to the CNS include the olfactory nerve, the trigeminal nerve, and the vascular and lymphatic systems. The OSN or olfactory nerve has axons that project to the olfactory bulb. These axons are encapsulated by layers of olfactory ensheathing cells (OECs) and olfactory neural fibroblasts (ONFs), which together form a perineural sheath [9]. This perineural space is physically continuous with the meninges of the brain. The trigeminal nerve is responsible for general sensations like pain and temperature and is distributed across both the respiratory and olfactory mucosa [11,13,64]. Its branches, specifically the V1 ophthalmic nerve and V2 maxillary nerve, are distributed in the nasal epithelium, with nerve fibers connecting directly from the nasal mucosa to the brainstem (pons). The lamina propria beneath the nasal mucosa is highly vascularized, promoting systemic absorption, and also contains lymphatic vessels. The olfactory/nasal lymphatic system provides a pathway for cerebrospinal fluid (CSF) to drain to the deep cervical lymph nodes, which can be exploited for drug delivery [9,62,65,66,67]. It has been reported that dural lymphatic vessels near various cranial nerve outlets at the skull base connect to deep cervical lymph nodes through nasopharyngeal lymphatics or via nasal and hard palate lymphatics [65,66]. Notably, the nasal mucosa is known to be composed of structures rich in lymphatic vessels and venous sinusoids [67].

### 5.2. Mechanisms of CNS Drug Delivery

Drugs administered intranasally access the CNS through two principal pathways, both allowing them to bypass the BBB.

#### 5.2.1. Intracellular/Intraneuronal Pathway

**Figure 2:**
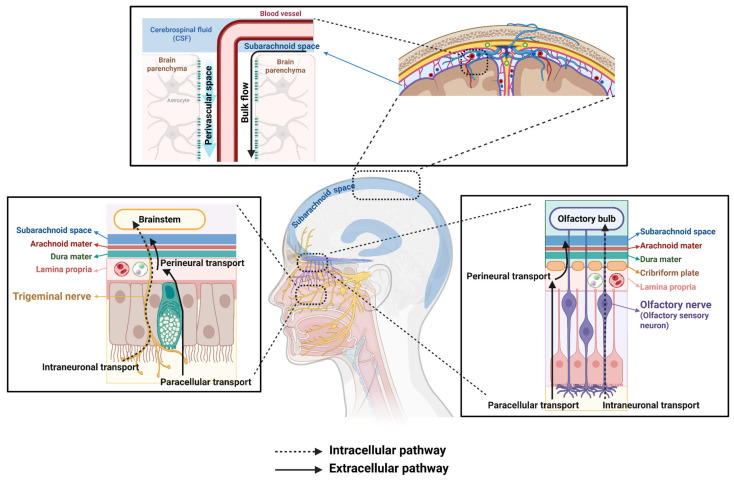
Illustration of nose-to-brain delivery mechanism.

This route involves drug uptake by peripheral nerve cells (olfactory or trigeminal) in the nasal mucosa, followed by transport to the brain via axonal transport (Figure 2).

##### Olfactory Nerve Pathway

Drugs are absorbed via endocytosis or pinocytosis by OSNs in the olfactory epithelium. The vesicles containing the drug travel along the OSN axon via anterograde axonal transport to the olfactory bulb [9]. Upon arrival, the drug is released into the synaptic cleft via exocytosis and can disperse to other cerebral areas via secondary neurons like mitral cells and tufted cells [9,12,63]. However, the calculated speed of axonal transport suggests the 4 mm axon length would require 0.84–2.7 h for transport [12,64]. This relatively slow timescale fails to explain the drug distribution observed within minutes in the brain [9,14,63]. Consequently, axonal transport is considered a secondary or auxiliary pathway for immediate drug distribution.

##### Trigeminal Nerve Pathway

This pathway involves drug uptake via endocytosis by the terminal processes of the trigeminal nerve branches (V1, V2), which extend just below the tight junctions in the respiratory and olfactory epithelia [9]. The drug then travels along the trigeminal nerve axon to the trigeminal nucleus in the pons of the brainstem. Given the greater anatomical distance in this route (e.g., 20 mm in rodents), axonal transport-mediated delivery is expected to be even slower [9,12,64]. This further reinforces the conclusion that intraneuronal axonal transport is not the principal mechanism underlying the rapid drug kinetics observed shortly after intranasal delivery.

#### 5.2.2. Extracellular/Extraneuronal Pathway

This pathway involves the drug moving directly to the CNS via spaces surrounding the cells, and it is strongly suggested to be the major mechanism for direct brain delivery (Figure 2).

##### Paracellular Transport: Crossing the Nasal Epithelia

Drugs move through the paracellular cleft by passing between epithelial cells, across the tight junctions (TJs), to reach the lamina propria [12]. The olfactory epithelium is relatively more permeable compared to other barriers like the BBB. The continuous turnover of OSNs can create open intercellular clefts where TJs temporarily loosen, allowing large molecules to pass [64,68]. Alternatively, highly lipophilic drugs can reach the lamina propria via passive diffusion across the cell membrane [12,14,63].

##### Perineural Transport

Once in the lamina propria, the drug enters the perineural space and sheath surrounding the olfactory nerve axon bundles [12] (Figure 2). Among the three meningeal layers, the arachnoid is generally known to act as the physical barrier of the meninges, since its arachnoid barrier cell (ABC) layer is well developed with tight junctions and adherent junctions [69,70]. However, several recent studies have reported that the arachnoid barrier covering the olfactory bulb differs from other ABCs, showing differences in the expression of molecules such as E-cadherin, and may therefore be more leaky [71,72]. These findings suggest that this region could represent a site for cerebrospinal fluid (CSF) outflow [71]. In other words, the olfactory region provides a space where the perineural sheath can directly connect to the subarachnoid space (SAS). Thus, drugs delivered via the intranasal route not only bypass the BBB but may also avoid the tightness of arachnoid barrier cells that is otherwise well developed in other brain regions, enabling more efficient delivery into the brain [15,16,73,74]. Numerous preclinical studies have demonstrated the efficacy of intranasal delivery approach for Alzheimer’s disease [16], migraine and chronic pain [73]. Furthermore, the trigeminal nerve is connected to the brainstem through this perineural space [9,75]. While CSF outflow through the trigeminal pathway has been reported [76], it remains unknown whether the meninges in this region are also characterized by particularly leaky arachnoid barrier cells.

##### Bulk Flow Through Perivascular Spaces (PVS)

Once the perineural space of the trigeminal nerve or olfactory nerve enters the brain, the most plausible mechanism for rapid distribution of drugs across wide brain regions is bulk flow through the perivascular space (PVS) (Figure 2). In this space, drug transport occurs not by diffusion, but by bulk flow or convection [9,12,73]. This mechanism is associated with the so-called “perivascular pump” driven by arterial pulsations induced by the cardiac cycle. Crucially, this convective transport provides the most convincing explanation for the rapid kinetics (within minutes) and widespread distribution observed experimentally. In contrast to the hours required for axonal transport, bulk flow through the PVS allows substances administered intranasally to quickly reach deep brain structures [12,64]. Indeed, imaging studies confirmed this rapid transit; fluorescently labeled dextran (3 kDa) administered intranasally was clearly observed in the PVS of cortical surface arteries and deeper vessels within 20 min. This evidence strongly supports the role of convective transport within the PVS as the critical and predominant mechanism. This transport is responsible for the rapid and widespread brain distribution achieved shortly after intranasal delivery [77]. In addition, insulin has also been confirmed to reach the brain via the perineural space along the trigeminal nerve [75].

### 5.3. Effectiveness of Intranasal Drug Delivery Compared to Conventional Routes

Intranasal (IN) drug delivery offers several advantages over conventional administration routes such as oral or intravenous injection. (1) Because IN delivery is non-invasive, patient compliance is improved. (2) This route provides a direct pathway that bypasses the blood–brain barrier (BBB) while also avoiding hepatic first-pass metabolism, thereby enhancing drug bioavailability in the brain and reducing the required dosage [73,78]. (3) Systemic side effects can be minimized; for instance, intranasal administration of deferoxamine (DFO) after stroke resulted in significantly higher concentrations in brain tissue while maintaining lower levels in blood and peripheral organs compared to intravenous delivery [79]. Consistently, recent studies employing circular RNA demonstrated greater accumulation in the peri-infarct region and reduced nonspecific distribution in other organs when administered intranasally rather than intravenously [80]. Furthermore, intranasal delivery of anti-Nogo-A antibodies achieved comparable concentrations in CNS tissue to those observed after intrathecal injection, highlighting its ability to reach therapeutic levels in the brain without invasive procedures [81]. (4) Finally, IN delivery provides rapid access to the CNS, with drugs detected in brain tissue within 5 min of administration and reaching more distant brain regions within 30 min [11,73], collectively underscoring its promise as an efficient and clinically valuable route for CNS drug delivery.

### 5.4. Translational Hurdles in Nose-to-Brain Delivery: Anatomical and Physiological Disparities

While nose-to-brain drug delivery presents distinct advantages over conventional administration routes, significant translation hurdles impede its clinical implication. The most significant and systemically overlooked anatomical discrepancy lies in the olfactory epithelium, the primary portal for the direct neuronal nose-to-brain (N2B) pathway. In rodents (rats and mice), this specialized region is expansive, constituting as much as 50% of the total nasal surface area [64]. In contrast, the human olfactory region is a small, remote patch located in the superior aspect of the nasal cavity, accounting for only approximately 10% of the total nasal surface area. This 5-fold difference in relative surface area implies a fundamental, qualitative difference in the dominant transport mechanism between the species. In a rodent model, a drug administered into the nasal cavity has a high probability of interacting with the massive olfactory epithelium, leading to efficient direct brain transport via the olfactory nerve pathway. In a human, however, 90% of the nasal cavity is lined with respiratory epithelium. Consequently, for any therapeutic administered via a standard nasal spray, the dominant outcome is not direct brain delivery. Instead, the drug is either rapidly cleared by mucociliary action or, more likely, absorbed into the systemic circulation via the highly vascularized respiratory mucosa, nullifying the primary BBB-bypassing advantage [12]. Therefore, what is observed as a high-efficiency, primary delivery route in rodent models is, in humans, very likely a low-efficiency, auxiliary pathway. Preclinical studies are thus systemically biased to overestimate the potential for direct brain transport.

In addition, significant differences exist in the structure of nasopharyngeal immune tissues. Rodents possess well-characterized, organized nasopharynx-associated lymphoid tissue (NALT), whereas humans have a more diffuse Waldeyer’s ring (comprising the lingual, pharyngeal, palatine, and tubal tonsils) [82]. This difference in immune architecture has profound implications for the translatability of N2B biologics, proteins, and cell therapies. These treatments may elicit vastly different immune responses and trafficking patterns in human versus rodents. Furthermore, there are tremendous differences in the lengths, volumes, and surface areas between the nasal cavities of adult humans and rodents. In addition, species-specific variations in mucosal permeability and metabolic processes (e.g., enzymatic degradation) complicate direct translation [83].

## 6. Intranasal Therapeutics for Ischemic Stroke: Evidence from Pre-Clinical Models

Numerous therapeutic agents, from small molecules to biologics and cell therapies, have been evaluated for intranasal delivery in preclinical stroke models (Table 1).

### 6.1. Small Molecule

#### 6.1.1. Lipid Derivative

Recent preclinical stroke research has focused on lipid derivatives as a promising class of neuroprotective agents. Elovanoids (ELVs), a new class of homeostatic lipid mediators derived from very long-chain polyunsaturated fatty acids (VLC-PUFAs), have shown efficacy in animal models of ischemic stroke and traumatic brain injury [84,111]. In particular, intranasal delivery of elovanoids precursors improved neurological deficit and reduced infarct volume [84]. Elovanoids precursors upregulated anti-inflammatory and pro-homeostatic genes, thereby effectively modulating neuroinflammation. Similarly, sodium butyrate (NaB), a short-chain fatty acid, also provides neuroprotection following intranasal delivery. This effect is mediated by the activating the Phosphatidylinositol 3-kinase (PI3K)/A serine/threonine-specific protein kinase (Akt) pathway via the G Protein-coupled Receptor 41 (GPR41)/Gβγ signaling cascade. This mechanism, driven by the ischemia-induced upregulation of GPR41 in neurons, ultimately attenuates neuronal apoptosis [85].

#### 6.1.2. Synthetically Derived Small Molecules

The potential of repurposed and novel small molecules for stroke treatment via the intranasal route has been a key area of investigation. One example is naloxone, a drug with over 50 years of clinical history as an opioid antagonist. Cases were reported long ago that intravenous injection of naloxone could show therapeutic effects in acute stroke patients [112,113]. While its (−)-enantiomer has a high-affinity for opioid receptors, the less active (+)-naloxone isomer was specifically investigated for its independent anti-inflammatory properties [112,113,114]. Recently, an in vitro study showed that naloxone can exert an anti-inflammatory effect by nonselectively blocking Toll-like Receptor 4 (TLR4) signaling in microglia [114]. When administered in a delayed fashion intranasally, this isomer was shown to significantly reduce neurological deficits and infarct volume. This effect is attributed to its ability to suppress microglial activation and subsequent Tumor Necrosis Factor α (TNF-α) secretion [86].

Concurrently, Edaravone, a potent scavenger of hydroxyl radicals, was first shown to have an anti-oxidant and anti-inflammatory role in ischemic stroke in mice [115]. In addition, edaravone dexborneol, a related formulation, has been reported to be clinically effective in acute ischemic stroke patients [116]. Recently, Edaravone-IL, a new ionic liquid formulation, provided superior neuroprotective effects, including a significant reduction in infarct volume and cerebral edema, compared to its conventional solution form [87].

A sophisticated approach was explored to deliver dexmedetomidine, a selective α2-adrenoceptor agonist used clinically as a sedative, anesthetic, and analgesic [117]. To overcome the challenges of systemic side effects from widespread receptor distribution and rapid nasal clearance, a novel multivalent bio-adhesive nanoparticle cluster was developed to ensure sustained release. The loaded DEX was neuroprotective by concurrently inhibiting apoptosis via the PI3K/Akt pathway, suppressing inflammation through the TLR-4/NF-κB pathway, and mitigating oxidative stress [88].

### 6.2. Protein and Peptides

#### 6.2.1. Neurotrophic/Growth Factors and Peptide Hormones

Brain-Derived Neurotrophic Factor (BDNF) is a key neurotrophic factor known for enhancing synaptic plasticity, promoting neuronal survival, and facilitating functional recovery in various brain injuries [118,119]. As a large protein, BDNF faces significant challenges in crossing the blood–brain barrier (BBB) and is prone to rapid in vivo degradation, limiting the clinical utility of direct protein administration [120,121]. To overcome these limitations, recent studies have pioneered innovative intranasal delivery strategies. Indeed, in neonatal hypoxic ischemia models, it has been shown that intranasal administration of BDNF can improve neural plasticity and neurobehavioral outcomes [121]. Building upon this principle, more advanced approaches have been explored. For instance, gene therapy approaches have utilized adeno-associated virus (AAV) vectors to deliver BDNF and its receptor, TrkB, via intranasal routes. These promoted synaptic plasticity and motor function recovery by enhancing corticospinal connections [89]. Another study demonstrated that intranasally delivered mesenchymal stem cells overexpressing BDNF also yielded superior, albeit temporary, functional recovery in a neonatal stroke model [122]. These findings collectively highlight intranasal delivery as a powerful, non-invasive method for achieving targeted brain delivery of BDNF and other neurotrophic factors.

Insulin-like growth factor-1 (IGF-1) is a polypeptide primarily synthesized in the liver in response to growth hormone, and it is known to regulate neurogenesis, myelination, and anti-apoptotic effects [123,124,125]. Clinically, low circulating IGF-1 levels are correlated with an increased stroke risk, higher mortality, and poorer functional outcomes, though results can be inconsistent [126]. Preclinical studies have consistently shown IGF-1’s potent neuroprotective and neuroregenerative effects, mitigating neurological deficits and improving behavioral outcomes [19,126]. While circulating IGF-1 can modulate brain cells by passing through the BBB, this process is often slow and inefficient due to the drug’s short half-life in the bloodstream. Thus, intranasal delivery of IGF-1 has been studied in preclinical stroke models, where it effectively improved neurobehavioral outcomes and reduced infarct volumes [90,91]. 75 μg [90] as well as 150 μg [91] administration three times in rats with MCAO was shown to be effective. More recently, intranasally delivered IGF-1 was co-administered with bone marrow mesenchymal stem cells (BMSCs) [109]. The treatment not only enhanced the survival and homing of the BMSCs in vitro but also significantly promoted neurogenesis and angiogenesis in vivo. This combined therapy led to superior recovery in motor and sensory functions compared to IGF-1 or BMSC treatment alone.

Growth factors whose expression is altered in response to ischemia have been explored as promising candidates for intranasally delivered therapeutics in preclinical stroke research. For example, mesencephalic astrocyte-derived neurotrophic factor (MANF) is a cytoprotective protein typically expressed in neurons under normal conditions [127,128]. MANF was shown to promote retinal repair in mice and drosophila [128]. Following injury, however, its expression shifts to microglia and macrophages. By administering exogenous MANF intranasally, researchers were able to reduce the expression of pro-inflammatory cytokines such as IL-1β, IL-6, and TNF-α, while simultaneously increasing anti-inflammatory cytokine IL-10. This resulted in a significant reduction in infarct volume and improved neurobehavioral outcomes [92]. Another example is Vascular Endothelial Growth Factor D (VEGFD). It is normally expressed in neurons and serves as a key regulator of dendritic integrity and cognitive function [129,130]. VEGFD expression is regulated by nuclear calcium; it mediates dendritic arborization [130] and plays a role in memory consolidation [129]. Following a stroke, the activation of extrasynaptic NMDA receptors leads to a decrease in VEGFD resulting in the loss of dendritic spine structure. The intranasal delivery of exogenous VEGFD was shown to counteract this loss, thereby preserving dendritic structure, reducing brain damage, and promoting neuroregeneration [93].

Another strategy to promote neuronal protection and regeneration is the exogenous administration of substances that regulate neurogenesis, with Wingless-Type MMTV Integration Site Family, Member 3A (Wnt3a) being a prime example. As a glycoprotein and a key regulator of the Wnt/β-catenin signaling pathway, Wnt3a is the first purified Wnt protein [131]. Wnt3a is essential for the self-renewal of neural stem cells and neural progenitor cells [132]. This role is well-documented in both developmental cortical neurogenesis [133] and adult hippocampal neurogenesis [134]. Following a stroke, a decrease in endogenous Wnt3a and β-catenin was observed in the subventricular zone, a key neurogenic region [94]. Researchers have found that the intranasal delivery of recombinant Wnt3a successfully activates the Wnt/β-catenin pathway, rescuing the ischemia-induced reduction of endogenous Wnt3a. This non-invasive approach not only increased proliferating neurons and promoted angiogenesis but also led to a significant recovery in cerebral blood flow and long-term functional recovery [94]. Another study highlighted Wnt3a’s neuroprotective role [95]. It showed that intranasal delivery of Wnt3a activates the Frizzled-1 (Frz1)/PIWI-like protein 1a (PIWI1a)/Forkhead box M1 (FOXM1) pathway, highlighting Wnt3a’s dual function in both protecting existing neural tissue and stimulating endogenous repair

#### 6.2.2. Chemokines/Cytokines

The recovery process following a stroke is a dynamically regulated process involving complex interactions among various immune cells, including neutrophils, microglia, monocyte-derived macrophages, and lymphocytes, as well as other glial cells like astrocytes and oligodendrocytes [2,6,27]. Given this intricate neuroinflammatory response, numerous cytokines have been explored as potential therapeutic targets to modulate this process. Among these, anti-inflammatory cytokines, particularly, interleukin-13 (IL-13) and Interleukin-4 (IL-4), delivered via the intranasal route, were reported to promote white matter recovery in ischemic stroke. IL-13 acts indirectly by inhibiting Signal Transducer and Activator of Transcription 3 (STAT3) phosphorylation, which polarizes microglia and macrophages toward an anti-inflammatory state to promote white matter repair [96]. In contrast, IL-4, key type 2 immunity cytokine has a more direct regenerative role [97]. It promotes oligodendrocyte differentiation and remyelination via the Peroxisome Proliferation-Activated Receptor γ (PPARγ) signaling axis, a mechanism critical for long-term white matter recovery. Interestingly, IL-4 has also shown a therapeutic effect in hemorrhagic stroke following intranasal delivery [135]. In this context, its primary therapeutic effect was mediated by the Signal Transducer and Activator of Transcription 6 (STAT6)/Suppressor of Tumorigenicity (ST2) pathway, which enhances the phagocytic capacity of immune cells for efficient blood clot clearance. These findings collectively demonstrate that anti-inflammatory cytokines can be used to elicit specific, targeted responses that address the unique pathophysiology of different stroke subtypes.

Recent preclinical research has explored a novel therapeutic approach by modulating innate immunity through the complement peptide C3a. Stokowska et al. initially demonstrated that delayed intranasal C3a administration, starting in the subacute phase, promoted neuroplasticity and long-term functional recovery without affecting infarct volume [98]. Subsequent work explicitly confirmed their hypothesis [99]: while C3a signaling may be detrimental in the acute phase of stroke, its action in the later phases is highly beneficial. They further unveiled a core mechanism by which C3a accelerates recovery. C3a administration modulated astrocyte reactivity and upregulated key neuroplasticity markers such as Igf1 and Thbs4. Moreover, they provided compelling evidence that C3a treatment enhances global white matter reconstruction and improves cortical connectivity, highlighting its unique role in stimulating structural repair and functional recovery after ischemic stroke.

Osteopontin (OPN), a multifunctional phosphorylated glycoprotein, has emerged as a promising therapeutic candidate for ischemic stroke due to its established roles in a variety of physiological and pathological processes. OPN is normally expressed at low levels in neurons and microglia [136]. Its expression is significantly upregulated in response to cerebral injury, suggesting a key endogenous protective function [137]. OPN has been suggested as an opsonin that aids phagocytosis by microglia and macrophages in stroke animal models [137]. Furthermore, it has been reported that OPN induces the migration of astrocyte and microglia in the striatum during acute injury [138]. Subsequent studies suggest that under various injury conditions, OPN adheres to the mitochondria of damaged neurons [139]. OPN also plays a crucial role in the formation of protein aggregates known as corpora amylacea [140,141]. In seminal study, OPN, particularly when cleaved by thrombin, showed robust neuroprotective effects when administered intranasally [100]. Subsequent studies sought to overcome the limitations of OPN peptides [101,102,103]. First, they encapsulated osteopontin in gelatin nanoparticles to extend the therapeutic window [101]. Later, they shortened the initial 20-aminoacid peptide into a more potent 7-amino acid version [102,103]. Mechanistically, this series of studies collectively revealed that OPN peptides act by binding to key integrin receptors [102]. This binding, in turn, modulates STAT1 phosphorylation, and promotes a beneficial shift in microglial phenotype toward an anti-inflammatory state. This anti-inflammatory polarization enhances the microglia’s ability to clear cellular debris through efferocytosis, showcasing OPN’s unique role in both mitigating inflammation and actively promoting a regenerative environment [103].

#### 6.2.3. Others

##### Tissue Plasminogen Activator (tPA)

Tissue plasminogen activator (tPA) is an established fibrinolytic agent for acute stroke. In a series of studies, it has been repurposed as a promising neurorestorative agent delivered intranasally. These approaches promote brain plasticity and functional recovery in the subacute and chronic phases, safely circumventing its hemorrhagic side effects. In 2018, Chen et al. first demonstrated this novel application [104]. They showed that intranasal tPA administered during the subacute phase (7 days post-stroke) did not reduce infarct volume but significantly enhanced sensorimotor function. This was achieved by promoting corticospinal tract axonal remodeling and neurite sprouting, confirming a neurorestorative effect independent of its clot-busting activity. Building on this, Pu et al. provided a more comprehensive mechanistic understanding [105]. They used tPA-deficient mice to show the critical role of endogenous tPA in long-term recovery. Most notably, they developed a protease-inactive mutant (tPA-S478A). This mutant, delivered intranasally, proved to be as effective as wild-type tPA in improving white matter integrity and promoting axonal sprouting. Crucially, it entirely eliminated the risk of intracerebral hemorrhage. This pivotal finding highlighted that tPA’s neurorestorative effects are protease-independent and mediated via Epidermal Growth Factor Receptor (EGFR) signaling. This paves the way for a safer, non-thrombolytic tPA-based therapy for stroke recovery.

##### Anti-Nogo-A Antibody

Neurite Outgrowth Inhibitor-A (Nogo-A) is known as a neurite growth-inhibiting and plasticity-restricting membrane protein that hinders axonal regeneration and plasticity following central nervous system (CNS) injuries [142,143,144]. The anti-Nogo-A antibody was first shown to improve motor function in spinal cord injured rats [144]. Later, a series of studies explored the therapeutic potential of anti-Nogo-A in promoting the restoration of corticospinal tracts after stroke in rats [142,143]. Correa et al. demonstrated that intranasal delivery of a monoclonal antibody against Nogo-A promoted axonal rewiring and significantly improved neurobehavioral outcomes at chronic stages of stroke (6 weeks) [81]. This research is particularly notable for showing that this non-invasive method can achieve therapeutic antibody concentrations in the CNS comparable to those of more invasive intrathecal injections. Furthermore, the delivery was well-tolerated and did not induce a major inflammatory response. This finding establishes intranasal delivery as a safe and effective alternative for chronic stroke treatment.

### 6.3. Nucleic Acid Therapy

Beyond small molecules and protein-based therapies, recent advances in nucleic acid medicine have enabled specific modulation of the complex gene networks involved in stroke. For instance, gene expressions can be modulated using tools like siRNA and Clustered Regularly Interspaced Short Palindromic Repeats (CRISPR). A study by Boutej et al. demonstrated that intranasal delivery of an siRNA targeting the RNA-binding protein SRSF3, a key regulator of the innate immune response in the brain [145], efficiently reduced SRSF3 protein levels in the brain. This reprogramming of innate immune response led to a significant reduction in the infarct volume. This was achieved by restoring the synthesis of critical immune proteins like Leukocyte Immunoglobulin-Like Receptor subfamily B member 4a (LILRB4a), Heme Oxygenase 1 (HMOX1), and Tissue Inhibitor of Metalloproteinases 1 (TIMP1). Similarly, Ryu et al. developed a protein-based CRISPR/dCas9 system to upregulate the expression of the neuroprotective gene sirtuin1 (Sirt1), a member of NAD+-dependent protein deacetylase [107,146]. Sirt1 knockout mice subjected to stroke displayed larger infarct volume [146]. Ryu et al. successfully enhanced Sirt1 expression through targeted intranasal delivery of dCas9-VP64 protein and its corresponding single-guide RNA (sgRNA) to the brain via the MCT1 transporter. This, in turn, mitigated secondary brain damage, reduced brain edema, and improved survival rates in mice [107].

**Figure 3:**
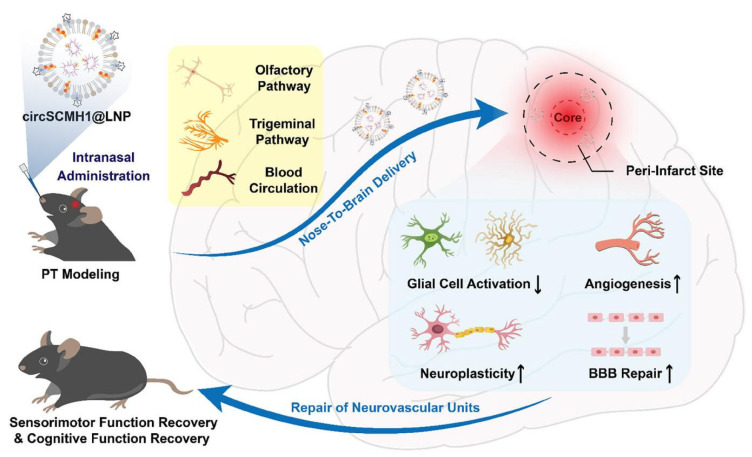
Schematic illustration of circSCMH1@LNP intranasal transport and improved post-stroke neurological function repair in an ischemic stroke mouse model. (Reproduced with permission from Ref. [80] 2025, Wiley-VCH GmbH).

In parallel, the direct delivery of therapeutic nucleic acids offers a distinct approach to compensate for the genetic dysregulation following brain injury. Circular RNAs (circRNAs), which are highly stable endogenous RNAs characterized by back-splicing and a closed-loop structure, and abundant in the CNS, have been studied for their restorative function in ischemic stroke. Among them, circular RNA form of Scm Polycomb Group Protein Homolog 1 (SCMH1) gene (circSCMH1), whose expression levels are decreased in the plasma of patients with acute ischemic stroke [147], was extensively studied in preclinical settings (Figure 3). Yang et al. (2020) reported that in rodents and nonhuman primates, intravenous (IV) delivery of circSCMH1 in extracellular vesicles (EV) promoted neuroplasticity and suppressed glial activation [147]. A following study by Li et al. demonstrated that IV delivery of circSCMH1 also enhanced vascular repair [148]. Building on these findings, intranasal delivery of circSCMH1 effectively bypasses the blood–brain barrier (BBB). The circRNA accumulated well within the peri-infarct region while minimizing off-target accumulation (Figure 3) [80]. The treatment significantly improved sensorimotor and cognitive function and reversed key pathological gene expression profiles.

### 6.4. Cell Therapy

Mesenchymal stem cell (MSC) therapy has emerged as a promising approach for treating ischemic stroke, with a shared focus on the cells’ powerful paracrine effects rather than direct cellular replacement [40]. To overcome limitations of conventional systemic delivery, such as unstable in vivo homing and reduced cell viability, researchers have increasingly explored intranasal delivery [149]. This approach has moved beyond simple cell administration, with studies attempting various methods to augment therapeutic efficacy and delivery efficiency to the target site.

#### 6.4.1. Strategies to Augment Therapeutic Efficacy

To enhance the therapeutic potential of MSCs, several strategies have been developed. Genetic modifications to overexpress neurotrophic factors like BDNF in MSCs were effective in reducing ischemic damage, as well as improving behavioral outcomes and the proliferation of newly generated cells [122]. In addition, intranasal delivery of bone marrow-derived MSCs (BMSCs) with hypoxia preconditioning showed a dramatic enhancement of homing and migratory effects [108]. Similarly, erythropoietin preconditioning was found to provide long-term functional benefits in neonatal stroke models [150]. Combination treatments have also proven effective; co-administering BMSCs with IGF-1 enhanced neurogenesis and angiogenesis, an effect mediated by increased migratory activity and reduced apoptosis of BMSCs [109]. Some studies have successfully visualized the direct homing of MSCs to the brain [108,109]. However, a recent trend, exemplified by Jiang et al., has shifted towards using cell-free derivatives like secreted factors and cell lysates [110]. These cell-free approaches have also yielded significant neuroprotection and improved functional recovery, further supporting the dominant role of the paracrine effect in this therapeutic paradigm [110].

#### 6.4.2. Comparison of MSC-Based Therapeutic Strategies

The evolution from using live MSCs to employing preconditioned cells or cell-free derivatives like extracellular vesicles (EVs; see Section 7.2 for details) reflects an effort to maximize therapeutic benefits while minimizing risks associated with cell transplantation. Each approach offers a unique profile of advantages and challenges, influencing its potential for clinical translation (Table 2).

#### 6.4.3. Translational Challenges and Considerations

Despite the promise of MSC-based therapies, several translational hurdles must be addressed, including the risks of vascular obstruction, immunogenicity, and the optimization of dosing strategies.

The risk of vascular obstruction is a significant concern, particularly with intravenous (IV) or intra-arterial (IA) administration of live cells. When administered intravenously, MSCs can become trapped in peripheral organs, especially the lungs, in what is known as the “pulmonary first-pass effect” [58,162]. This not only reduces the number of cells reaching the brain but also carries a risk of pulmonary embolism, which has been reported after IV infusion of adipose-derived MSCs [163]. Intra-arterial delivery can bypass the lungs but introduces the risk of cerebral infarction or micro-embolism if cells aggregate in cerebral arteries [164]. This risk is influenced by cell size, dose, and infusion speed [57]. Cell-free derivatives like EVs, being much smaller than whole cells, largely circumvent the risk of vascular occlusion, offering a significant safety advantage [165]. This highlights the strategic advantage of the intranasal route. By bypassing the systemic vasculature entirely, intranasal administration inherently avoids both the pulmonary first-pass effect and the risk of embolism associated with intravascular injection. This positions it as a particularly safe delivery platform for both cell-based and cell-free therapeutics.

Immunogenicity is another critical consideration for cell-based therapies. A key advantage of MSCs is their inherently low immunogenicity, which allows for allogeneic transplantation, often without the need for immunosuppressive drugs [58]. Clinical studies have generally reported a good safety profile in this regard, with no instances of graft-versus-host disease in some trials [40]. Similarly, MSC-derived EVs possess immunomodulatory properties that can help mitigate inflammatory responses [159]. Future strategies to further reduce immune rejection include genetic engineering (e.g., CRISPR/Cas9) to create “hypoimmunogenic” cells or encapsulating cells in protective biomaterials [166].

Finally, the administration frequency is a key variable for therapeutic efficacy. Preclinical studies suggest that repeated administration of MSCs can yield superior outcomes compared to a single dose [167]. For instance, one study found that dual doses at 8 and 24 h post-stroke were more effective than a single 24 h dose [168]. One clinical trial has also explored repeat-dosing regimens, involving multiple injections over several weeks [169]. In this context, the intranasal route is particularly advantageous as its non-invasive nature facilitates delayed and repeated administrations, which has been shown to enhance regeneration and functional recovery [167].

#### 6.4.4. Clinical Trial of Intranasal Stem Cell Therapy

The clinical translation of mesenchymal stem cell (MSC) therapy for stroke has recently advanced with a focus on perinatal arterial ischemic stroke (PAIS) in neonates. The first-in-human, single-arm, open-label PASSIoN study (Perinatal Arterial Stroke treated with Stromal cells IntraNasally) assessed the feasibility, safety, and preliminary efficacy of intranasal (IN) delivery of allogeneic human bone marrow-derived MSCs [170,171]. The initial report demonstrated the treatment’s acute and subacute safety, showing that a single dose was well-tolerated with no serious adverse events or unexpected structural brain abnormalities on MRI [170]. The protocol for rapid diagnosis and treatment within seven days of symptom onset was also proven to be feasible. A subsequent report provided long-term follow-up at the two-year mark, showing continued safety and, importantly, promising exploratory efficacy [171]. Compared to a non-MSC-treated registry cohort, the MSC-treated infants showed a significantly lower incidence of cerebral palsy, better gross motor function, and an earlier age of independent walking. Furthermore, MRI findings at three months indicated less asymmetry in key motor tracts and less tissue loss. This strongly suggests that IN MSC therapy may offer a new and effective neuroprotective and regenerative intervention to improve long-term outcomes for neonates with PAIS.

### 6.5. Clinical Feasibility and Timeframe Considerations for Intranasal Therapeutics

The preclinical studies summarized above demonstrate the potential of various intranasally delivered agents across different phases of ischemic stroke. Small molecules targeting acute mechanisms like oxidative stress (e.g., Edaravone-IL) or excitotoxicity, along with early-acting anti-inflammatory agents (e.g., NaB, possibly (+)-Naloxone depending on timing), are primarily relevant for acute neuroprotection. These small molecules are ideally administered within hours of stroke onset. This timeframe aligns with the existing window for reperfusion therapies, suggesting potential combination strategies. However, this requires rapid diagnosis and administration capabilities in clinical settings. Repurposed drugs like naloxone may have an accelerated path to clinical trials due to existing safety data.

In contrast, agents promoting neurogenesis, angiogenesis, white matter repair, and neuroplasticity, such as growth factors (BDNF, IGF-1, Wnt3a), certain cytokines (IL-4, IL-13), complement peptides (C3a), repurposed tPA, anti-Nogo-A antibodies, and nucleic acid therapies (circSCMH1), are generally administered in the subacute to chronic phases (days to weeks post-stroke) in preclinical models. These target the brain’s endogenous repair mechanisms and aim to improve long-term functional recovery. Their delayed administration window is clinically more feasible for many patients who miss the hyperacute treatment window.

However, translating these findings faces significant hurdles. Biologics (proteins, antibodies) often have poor stability, complex manufacturing processes, and potential immunogenicity, although intranasal delivery aims to minimize systemic exposure. Nucleic acid therapies face similar challenges regarding stability, delivery efficiency into target cells, and potential off-target effects or immunogenicity [172]. Cell therapies (MSCs) present unique challenges including manufacturing scale-up under GMP conditions, ensuring batch consistency, cell viability post-administration, potential immunogenicity (for allogeneic cells), and long-term safety (e.g., tumorigenicity, although low for MSCs) [82]. While the PASSIoN trial demonstrated feasibility in neonates, translating this to the adult population requires addressing anatomical differences and potentially different dosing or delivery strategies. The success of these subacute/chronic therapies hinges not only on demonstrating efficacy in robust preclinical models (including those with comorbidities) but also on developing scalable manufacturing processes and establishing long-term safety profiles.

## 7. Nanotechnological Approaches for Treating Stroke via Nose-to-Brain Route

While intact MSC or cell lysate-based therapy have been utilized for intranasal stroke therapeutics, drawbacks such as low delivery efficiency, high production costs, and complex isolation procedures remain significant hurdles. Consequently, cheaper and controllable synthetic methods using simpler compositions are drawing interest, particularly for therapeutics such as small molecules, drug-loaded nanoparticles, and liposomes. However, most small molecule drugs show fast clearance, limited targeting efficiency, and low bioavailability.

To overcome these limitations, nanoparticle-based therapeutics (NBTs) have been developed (Table 3). NBTs offer a prolonged half-life, high drug loading efficiency, and improved targeting through both active and passive mechanisms. The tunable properties of NBTs are particularly advantageous for intranasal delivery; their efficacy is determined by factors like size, surface functionalization, composition, and even phase transition. This tunability is critical for penetrating the tight barriers of the nasal mucosa. For example, cell-penetrating moieties can be tethered to NBTs to further enhance cytosolic drug delivery.

This nanoparticle-based approach is especially relevant for CNS disorders. With conventional intravenous administration, nanoparticles or nanocomposite-based systems still face the unavoidable obstacle of the blood–brain barrier (BBB), whose highly restrictive structure limits efficient delivery to the brain. In contrast, intranasal delivery provides an alternative route bypassing the BBB and allows efficient drug transport, particularly for nanoparticle-based systems. While intranasally administered therapeutics may also reach systemic circulation via the respiratory tract, our focus here is on the direct nose-to-brain pathway as discussed in Section 5 [7]. The specific nanomaterial-based therapeutics discussed in the following sections are summarized in Table 4.

### 7.1. Hyaluronidase-Assisted Cell-Based Therapy

Hyaluronidase, an enzyme of approximately 55–61 kDa with a diameter of ~3 nm, has been used as a pretreatment in ischemic stroke models to loosen the extracellular matrix by degrading hyaluronic acid (HA). By reducing structural barriers, hyaluronidase enables more efficient delivery of the drugs and MSCs to target regions of the brain [173]. Intranasal administration of hyaluronidase can increase nasal mucosal permeability and facilitate the entry of therapeutic agents, such as stem cells or nanoparticles, into the CNS while bypassing the BBB [174]. However, dosing must be carefully controlled: while a dosing range of approximately 100 U per mouse is recommended, excessive amounts can cause acute inflammation and anaphylaxis.

Building on the preclinical evidence for MSC-based therapies described in Section 6, recent efforts have focused on nanotechnology and pretreatment strategies to enhance the efficiency of intranasal delivery. A report by Chau et al. showed that intranasal administration of BMSCs, when combined with hyaluronidase pretreatment 30 min prior to delivery, markedly improved therapeutic outcomes in ischemic stroke [167]. In this study, hypoxic-preconditioned BMSCs (HP-BMSCs) were employed, which exhibited enhanced survival potential and robust migration to the peri-infarct cortex. The rats showed significant increase in angiogenesis, neurogenesis and overall functional recovery. Mechanistically, expression ratios of HIF-1α/18s and Bcl-xL/18s were elevated 3–5 fold compared with controls. With a similar concept, another study demonstrated that hyaluronidase pretreatment also enhanced the intranasal delivery efficiency of BMSCs when co-administered with IGF-1 [109]. More recently, Yamamoto et al. extended this approach to multilineage-differentiating stress-enduring (Muse) cells, isolated from human bone marrow using stage-specific embryonic antigen-3 (SSEA-3) antibody [175]. Following hyaluronidase pretreatment, mitochondria-positive Muse cells demonstrated engraftment near the infarct core and showed improved sensorimotor function, whereas only a marginal number of BMSCs were detected in control animals.

### 7.2. EV-Based Therapy

Extracellular vesicles (EVs) are nano-sized, lipid bilayer-enclosed particles released by nearly all cell types into the extracellular space. They facilitate intercellular communication by transferring active biomolecules, including proteins, lipids, and nucleic acids. EVs can be derived from the MSCs and other autologous cells. After multiple homogenization, purification, and filtering steps, EVs can be prepared as particles a few hundred nanometers in diameter. EVs contain lipids and proteins of cellular origin, which may lower their immunogenicity.

However, EV-based therapies face significant translational challenges. First, large-scale production remains a major bottleneck. The number of MSCs and autologous cells is often insufficient to produce sufficient quantities of EVs for therapeutic use. While other types of allogenic cells have been used to solve this, this approach induces the possibility of immune responses. Furthermore, the dose-dependent safety of EV-based therapeutics must also be studied, given their cargo of endogenous proteins or loaded drugs.

Second, the instability of EVs is another critical factor to consider for intranasal therapeutics. When administered intranasally, intact EVs are vulnerable to physical clearance (mucociliary clearance and drainage), enzymatic degradation (by proteases and nucleases), and the impermeability of nasal mucosa. These factors can lead to the rapid disintegration of the vesicles into their individual components [176].

**Figure 4:**
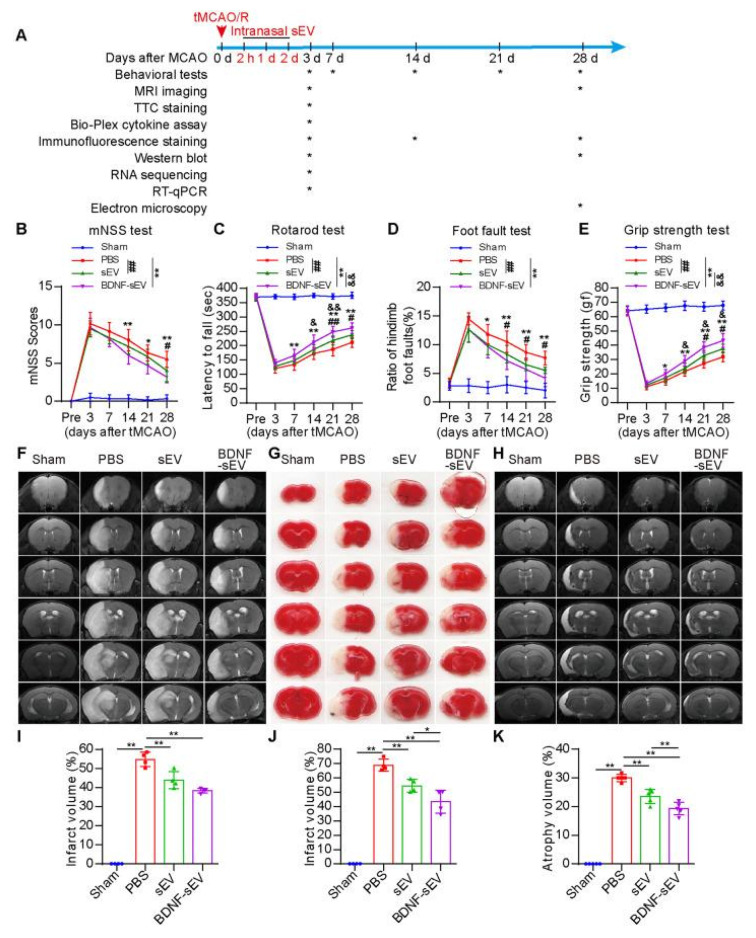
Functional improvement infarct volume reduction after the treatment of sEVs and BDNF-sEVs in tMCAO mice. (**A**) Illustration of the experimental timeline. (**B**–**E**) Behavioral tests, including mNSS scores (**B**), rotarod tests (**C**), foot fault tests (**D**), and grip strength tests (**E**), performed before and after injury. (**F**–**H**) Representative images of brain MRI (**F**,**H**) and TTC-stained coronal sections (**G**). (**I**–**K**) Statistical analysis of infarct volumes (**I**,**J**) and atrophy volumes (**K**). Statistical notations in (**B**–**E**): BDNF-sEV vs. PBS: * *p* < 0.05, ** *p* < 0.01; sEV vs. PBS: # *p* < 0.05, ## *p* < 0.01; BDNF-sEV vs. sEV: & *p* < 0.05, && *p* < 0.01. Statistical notations in (**I**–**K**): * *p* < 0.05, ** *p* < 0.01. (Reproduced with permission from Ref. [177] 2023, Elsevier.).

As discussed in Section 6, BDNF has emerged as a potential neurotrophic factor for ischemic stroke but faces major translational hurdles due to its large molecular size and instability in systemic circulation. Recent nanotechnology-based strategies have sought to address these issues by developing optimized carriers for intranasal delivery. One approach involves packaging BDNF into small EVs derived from genetically engineered MSCs [177] (Figure 4). Using lentivirus packaging, BDNF was overexpressed on iPSC-derived MSCs, and small EVs (BDNF-sEVs) were purified by anion chromatography. In a transient middle cerebral artery occlusion (tMCAO) model, BDNF-sEVs (50–150 nm) were delivered via the intranasal route following hyaluronidase pretreatment. BDNF-sEVS promoted sustained activation of BDNF/TrkB signaling, and ultimately improved functional recovery. Nonetheless, the mass production of BDNF-sEVs remains a significant challenge for future clinical applications.

Similarly, BMSC-derived EVs (BMSC-EVs) were tested in a tMCAO mouse model without hyaluronidase pretreatment [165]. Intranasal delivery of 150 nm BMSC-EVs resulted in modest functional recovery. These results highlight that both cellular origin of EVs and the delivery route critically influence therapeutic efficacy in ischemic stroke. In another study, ProtheraCytes, human CD34+ stem cells, were treated in an MCAO mouse model via three different routes: intracerebral (IC), intranasal (IN), and intra-arterial (IA) administration [178]. IC and IA routes produced superior recovery compared with IN in terms of infarct volume and sensorimotor functions. However, neurogenesis and angiogenesis markers were expressed at comparable levels across all delivery routes.

### 7.3. Liposome-Based Therapy

Liposomes are artificial vesicles composed of synthetic lipid bilayers, while extracellular vesicles (EVs) are naturally secreted by cells. This fundamental difference leads to key distinctions between liposomes and EVs. Liposomes are easier to scale up and allow higher drug-loading capacity. However, EVs are more biocompatible, naturally target tissues, and exhibit lower immunogenicity. Due to these complementary strengths, researchers are developing hybrid vesicles that combine the advantages of both systems [179]. In most cases, liposomes demonstrate longer circulation times than EVs because of their structural stability.

Basic fibroblast growth factor (bFGF) has been demonstrated as neuroprotective proteins in ischemic stroke mouse models when delivered via intracerebroventricular infusion. However, as a 16.5 kDa protein, bFGF does not cross the BBB efficiently, making systemic administration (intravenous, intraperitoneal, or subcutaneous) largely ineffective [180]. To improve delivery, the authors synthesized 100~130 nm bFGF-loaded liposomes (bFGF-NL) and injected them via the intranasal route. The bFB-NL treatment significantly improved sensorimotor function and reduced the infarct volumes in ischemic stroke mouse models [180].

Circular RNAs (circRNAs), already shown in preclinical models to promote neuroplasticity and vascular repair (see Section 6 and Figure 3), has been further optimized through encapsulation in lipid nanoparticles (LNPs) [80]. By incorporating PEGylated lipids, ionizable lipids, DSPC, and cholesterol, researchers generated a circSCMH1@LNP formulation suitable for intranasal administration [80]. When combined with hyaluronidase pretreatment, this system achieved efficient accumulation in the peri-infarct region while minimizing peripheral distribution. Beyond enhancing delivery efficiency, the liposomal strategy facilitated multiple reparative processes—including angiogenesis, synaptic remodeling, BBB stabilization, and myelin regeneration. These processes ultimately resulted in significant functional recovery in ischemic stroke models.

Recently, intranasal delivery of interleukin-4 (IL-4)-loaded lecithin-based liposomes was shown to enhance white matter integrity and attenuate long-term sensorimotor and cognitive deficits in MCAO models [97]. IL-4 promoted the structural and functional robustness of myelinated fibers, stimulated the differentiation of oligodendrocyte progenitor cells (OPCs) into myelinating oligodendrocytes and markedly improved long-term neurological outcomes.

The long-term circulation of the macrophage-mimetic liposomes can be achieved through the camouflage effect of the lipid components derived from the endogenous macrophages. A recent study tested liposomes co-loaded with panax notoginseng saponins (PNS) and ginsenoside Rg3 (Rg3), incorporated into macrophage-mimetic core–shell nanocomposites (MM-Lip-Rg3/PNS) [181]. These 150~180 nm nanocomposites preferentially targeted ischemic regions following intranasal administration, where they significantly reduced infarct volume, improved neurological function, and decreased pro-inflammatory cytokines (TNF-α, IL-6, and IL-1β). However, the precise mechanisms underlying the transport of most of the liposomal systems during intranasal delivery remain unclear.

### 7.4. Inorganic Nanoparticle-Based Delivery Vehicle

Intranasal drug delivery using inorganic nanoparticles has been demonstrated in several preclinical studies. With appropriate surface modifications and synthetic methods, gold nanoparticles (GNPs) can load antigen, drugs, and enzymes at the surface of GNPs and be delivered to the ischemic brain in mouse models. In one study, an ApoE-mimetic peptide (CS15) was covalently conjugated carboxylate thiol-PEG ligands on the surface of GNPs (CS15-GNPs) [182]. Following intranasal administration, 40 nm CS15-GNPs with a near-non-charged surface promoted recovery in the infarcted regions. Treatment with CS15-GNPs significantly suppressed neuronal apoptosis and cerebral inflammation, while no evidence of toxicity or accumulation of CS15-GNPs was observed.

**Figure 5:**
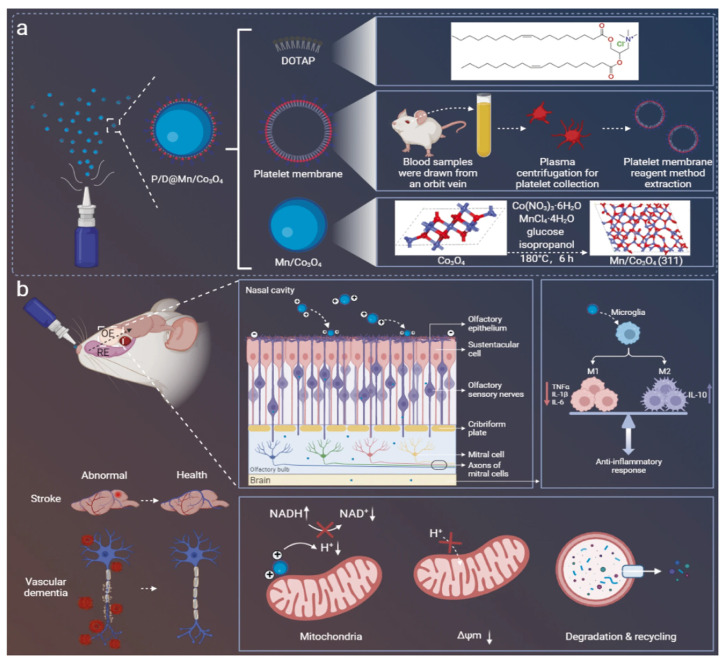
Synthesis, modification, and action mechanism of a nanotherapeutic agent in the amelioration of acute and chronic cerebral ischemic symptoms. (**a**) Synthesis of P/D@Mn/Co_3_O_4_. The process involves preparing Mn/Co_3_O_4_, extracting platelet membranes, and combining them with 2,3-(dioxy propyl)-trimethylammonium chloride (DOTAP). (**b**) In vivo mechanism of action. The agent is delivered intranasally and enters the brain via trigeminal and olfactory pathways. The platelet membrane targets inflammation, while the positive charge from DOTAP enhances mitochondrial accumulation. The particle’s enzyme-like activity facilitates reactive oxygen species (ROS) scavenging, reduces oxidative stress, and prevents neuronal apoptosis by depleting H^+^ around the mitochondria and inducing membrane depolarization. (Reproduced with permission from Ref. [183] 2023, Nature).

Platelet-mimetic membrane-coated Mn/Co_3_O_4_ nanoparticles were also delivered intranasally [183] (Figure 5). These 300 nm particles effectively scavenged reactive oxygen species (ROS) and induced M2 polarization of macrophages. This shift in M1/M2 balance resulted in decreased expression of pro-inflammatory cytokines (TNF-α, IL-6, and IL-1β) and increased expression of the anti-inflammatory cytokine IL-10.

Calcium phosphate (CaP) NPs have been widely studied as vehicles for drug and gene delivery due to their biocompatibility, biodegradability, and chemical similarity to the human bone. Researchers synthesized CaP NPs loaded with nucleotides (AMP, ADP, ATP and GTP) by mixing calcium solutions with each nucleotide (NT) solution [184]. To enhance intracellular uptake, branched polyethyleneimine (bPEI, 1.8 kDa) was coated onto the surface of nucleotide-loaded CaP NPs. Because sufficient nucleotide supply is required for recovery of ischemic stroke, intranasal delivery of NT-loaded CaP NPs reduced infarct volume and improved neurological deficits. Although the negative charge of ATP reduced its delivery efficiency, bPEI-tethered ATP loaded CaP NPs facilitated intracellular delivery.

In addition, Cas9 protein-loaded CaP NPs were intranasally delivered and shown to upregulate SIRT1 expression without acute cytotoxicity [107]. Even without hyaluronidase pretreatment, Cas9 protein successfully reached the infarcted brain region. The greatest increase in Sirt1 mRNA expression was achieved by tethering PEGs and PEIs onto the surface of Cas9-loaded CaP NPs.

**Figure 6:**
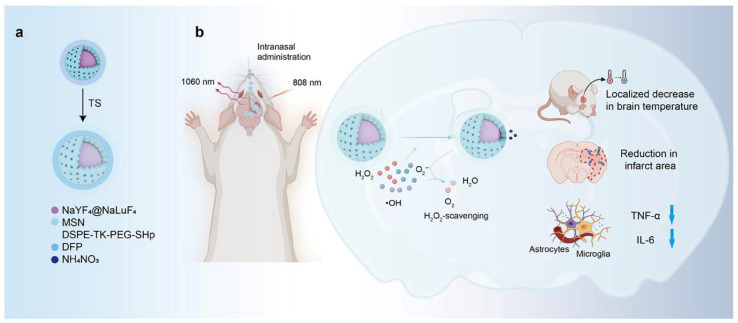
Schematic representation of the protective mechanism of cerebral ischemia-targeted and ROS-responsive nanocomposite (NMNP-TS) against cerebral ischemia–reperfusion injury in mice with transient middle cerebral artery occlusion (tMCAO) model. (**a**) The NMNP core is encapsulated by the ROS-responsive targeting polymer DSPE-TK-PEG-SHp (TS) to form the nanocomposite. (**b**) Following intranasal administration, NMNP-TS migrates to the ischemic site. The release of the refrigerant NH_4_NO_3_ from the nanocomposite locally reduces brain temperature, resulting in a therapeutic hypothermia effect, while the core scavenges reactive oxygen species. (Reproduced with permission from Figure 1 of [148], 2025, Wiley-VCH GmbH).

Wang et al. further demonstrated theranostic applications using rare-earth element-doped near-infrared (NIR, ~1060 nm) emissive NPs. These elements allowed in vivo imaging of superior tissue penetration depth, while also promoting recovery from ischemic injury [185] (Figure 6). Moreover, a stroke-homing peptide (SHp) was conjugated to polyethylene glycol (PEG) and 1,2-distearoyl-sn-glycero-3-phosphoethanolamine (DSPE) at the terminus of the PEG. The amphiphilic polymer, DSPE-TK-PEG-SHp (TS), was synthesized by employing a ROS-cleavable thioketal (TK) as the intermediate chain to connect DSPE and PEG. The TS surface encapsulated 100 nm sized NaYF_4_:Nd@NaLuF_4_@MSN@NH_4_NO_3_ (NMNP-TS), which scavenged hydrogen peroxide, reduced infarct size, and suppressed pro-inflammatory cytokines (TNF-α, and IL-6). However, the precise delivery pathways of inorganic NPs during transportation from the nasal cavity to the brain remain to be fully elucidated.

### 7.5. Oligomer/Polymer-Based Organic Compounds

Organic compounds have been developed for intranasal delivery for decades. FDA-approved polymers and surfactants have been widely used in this context. The use of organic polymers in intranasal drug delivery has been shown to enhance drug absorption and direct pharmaceutical agents to specific targets such as the brain. Commonly used organic polymers include natural polysaccharides such as chitosan and starch, as well as cellulose derivatives like hydroxypropyl methylcellulose (HPMC). Synthetic polymers, including poly(lactic-co-glycolic acid) (PLGA) and polyacrylates, have also been extensively applied due to their mucoadhesive and gel-forming properties.

Joachim et al. reported osteopontin (OPN)-loaded gelatin NPs (OPN-GNPs) could be used as a treatment for ischemic stroke [101]. Following intranasal delivery of OPN-GNPs in a middle cerebral artery occlusion (MCAO)-induced ischemic stroke, a significant reduction in infarct volume was observed. The same research group further reported the use of gelatin nanoparticles for the delivery of inducible nitric oxide synthase (iNOS) siRNA to the post-ischemic brain [186].

Reactive oxygen species (ROS)-responsive nanocarriers (SPNP) that can target mitochondria have been demonstrated to promote neurological functional recovery [187]. Puerarin (PU), an isoflavone from kudzu root, exerts anti-inflammatory, antioxidant, and lipid-lowering effects, and has shown therapeutic potential in metabolic, cardiovascular, hepatic, and neurological disorders. It is clinically approved in China for the treatment of myocardial ischemia. However, its poor solubility limits broader applications. To overcome this limitation, researchers developed a PU-loaded SPNP (chitosan-based polysaccharides NPs) formulation, which enables efficient delivery of small-molecule drugs into the brain. Intranasal delivery of PU-loaded SPNPs significantly reduced oxidative stress and pro-inflammatory cytokine levels and inhibited neuronal apoptosis.

The interaction between small-molecule drugs and cyclodextrin (CD) is characterized by a high degree of host–guest stability, making it well-suited for sustained drug release. In a recent study, Teng et al. reported a temperature-dependent sol–gel transition of drug-loaded NPs, which has significant implications for the treatment of ischemic stroke [188]. The hydrophobic pocket within CDs has been utilized as an inclusion site for borneol, a compound that has demonstrated efficacy in the treatment of inflammation- and oxidation-related diseases. In the experimental setup, poloxamer 407 and edaravone (EDA), a free radical scavenger, were co-loaded with borneol-loaded CD (BP) to form the EDA-BP sol. The solution can be injected into the nasal cavity, where it undergoes a phase transition. Injected solution transforms into a hydrogel upon contact with body temperature. The sol–gel transformation has been shown to enhance drug bioavailability. Intranasal delivery of EDA-BP significantly reduced infarct volume and improved neurological outcomes.

Similar to poloxamer 407, ionic liquids (ILs) have been applied for EDA (edaravone-IL) in ischemic mice [87]. Among these, ethoxy-ILs showed the highest cell viability and radical scavenging activity. Following intranasal administration of edaravone-IL, EDA was detected in the brain, olfactory bulb, nasal mucosa, and plasma. This formulation demonstrated exceptional therapeutic efficacy by reducing infarct volume and improving sensorimotor function.

Polymer-based drug delivery platforms have been developed for the treatment of ischemic stroke. Polylactic acid-hyperbranched polyglycerol (PLA-HPG) has been used as a surfactant to synthesize dexmedetomidine (DEX)-loaded NPs, which were further conjugated with the amines of dendritic polyamidoamine (PAMAM) to form a Schiff base (BNP-PAMAM/DEX) [88]. A Schiff base is an organic compound characterized by an azomethine group (–C=N–), a carbon-nitrogen double bond formed by the condensation of a primary amine with an aldehyde or ketone. This azomethine group can be broken into two different parts under slightly acidic conditions, releasing DEX into the olfactory bulb region. Intranasal administration of BNP-PAMAM/DEX resulted in the passive diffusion of DEX. It reduced neuronal apoptosis and decreased the expression of IL-6 and TNF-α, thereby providing neuroprotection.

**Table 4:** Summary of nanomaterial-based intranasal therapeutics for ischemic stroke. (N/A: not available).

Delivery Modality/Pretreatment	Drug/Active Cargo (Loading Dose)	Size/Charge	Outcomes	Reference
Hyaluronidase	hypoxic-preconditioned BMSCs	N/A	↑ Cerebral blood flow ↑ Neurogenesis	[167]
Hyaluronidase	BMSC + IGF-1	N/A	↑ Neurovascular regeneration ↑ Cerebral blood flow ↑ Sensorimotor functional recovery	[109]
Hyaluronidase	Multilineage-differentiating stress-enduring (Muse) cells	N/A	↑ Sensorimotor function recovery	[175]
MSC derived EVs/Hyaluronidase	BDNFs (188.8 ± 2.92 pg in 1 × 10^10^ EVs)	50~150 nm	↓ Infarct volume ↑ Sensorimotor functional recovery ↑ Angiogenesis	[177]
BMSC derived EVs/Hyaluronidase	EVs	~150 nm	↑ Sensorimotor function recovery	[165]
MSC derived EVs	CD34+ stem cells	86.7 ± 10.17 nm	↓ Infarct volume ↑ Sensorimotor function recovery	[178]
Protein-Liposomes	bFGF (3.33 mg/mL)	128 ± 7.65 nm/−15.3 ± 1.6 mV	↓ Infarct volume ↑ Sensorimotor function recovery	[180]
Circular RNA-Liposomes	circSCMH1 RNAs	140~170 nm/−1.15~–3.2 mV	↑ Sensorimotor functional recovery ↑ Cognitive functional recovery ↑ Angiogenesis	[80]
IL-4 loaded lecithin liposomes	IL-4 (5–100 ng/mL)	122.6 ± 2.4 nm/4.05 ± 1.35 mV	↓ Infarct volume ↑ Sensorimotor functional recovery ↑ White matter integrity	[97]
Macrophage mimetic liposomes	PNS, Rg3 (3–100 µg/mL)	177.7 ± 0.70 nm/–21.5 ± 1.11 mV	↑ Sensorimotor function recovery ↓ inflammation	[181]
Gold NP	ApoE-mimetic peptide (10 µM)	38.12 ± 1.7 nm/−4.46 ± 1.6 nm	↓ Infarct volume, ↓ Cerebral inflammation ↑ Sensorimotor function recovery	[182]
Mn-doped Co_3_O_4_ NPs		~300 nm/~7 mV	↓ Neuronal apoptosis, ↓ inflammation	[183]
NTP-Calcium phosphate NP	Nucleotides (240~300 µM)	10~20 nm/16~34 mV	↓ Infarct volume ↑ Sensorimotor function recovery	[184]
Cas9 loaded Calcium phosphate NP	Cas9 protein	43 nm/3.2 mV	↓ Cell death	[107]
UCNPs	Thioketal based ROS scavenger	105.7 nm/−7.3 mV	↓ Infarct volume, ↓ Inflammation ↑ Sensorimotor function recovery	[185]
Gelatin NPs	nitric oxide synthase (iNOS) siRNA (50–250 µg/kg)	100~300 nm	↓ Infarct volume ↑ Sensorimotor function recovery	[186]
Chitosan	Puerarin (6.25~100 µM)	32.82 ± 1.32 nm/16.0 ± 3.1 mV	↓ oxidative stress, pro-inflammatory cytokine ↓ Neuronal apoptosis,	[187]
Poloxamer 407, Cyclodextrin	Edaravone (10 mg/mL), Borneol (5 mg/mL)	N/A	↓ Infarct volume ↑ Sensorimotor function recovery	[188]
Ethoxy-Ionic liquids	Edaravone (20–200 µg/mL)	182.4 ± 43.2 nm/−38.6 ± 4.3 mV	↓ Infarct volume ↑ Sensorimotor function recovery	[87]
Polylactic acid-polyamidoamine complexes	Dexmedetomidine (0.675 mg/mL)	129.4~136.2 nm/−33.61 ± 1.3 mV	↓ Neuronal apoptosis, ↓ inflammation	[88]

## 8. Pitfalls and Future Directions

Despite its promise, intranasal (IN) drug delivery for ischemic stroke faces several important challenges that limit its translational potential. Anatomical and physiological barriers limit absorption efficiency [14,189]. These barriers include rapid mucociliary clearance and short residence time in the nasal cavity. Dosing capacity is also restricted by a small volume limit (approximately 100–200 μL per nostril in adults). In addition, only a small fraction of the nasal surface area corresponds to the olfactory region. This makes targeted delivery difficult and potentially leads to nonspecific absorption into the respiratory region with consequent systemic side effects [14,15,190]. Enzymatic degradation by proteases, esterases, and cytochrome P450 isoforms in the nasal mucosa, together with the presence of efflux pumps such as P-glycoprotein, further reduce drug stability and brain bioavailability [14]. Beyond these physiological and anatomical hurdles, safety and toxicity concerns must also be addressed. Repeated and excessive IN dosing, in particular, poses a risk. This can cause mucosal irritation or even damage to olfactory neurons. Furthermore, the nasal cavity’s large, highly vascularized surface area means that therapeutics can enter the systemic circulation, not just the brain. This systemic exposure, or even local activation of dendritic cells and macrophages in the nasal cavity, can be triggered depending on the injection volume, dosage, and frequency. Consequently, rapid CNS exposure or repeated administration could provoke immune perturbation, neurotoxicity, or excessive immune responses such as allergic reactions [14,82]. Nanocarriers themselves may also present distinct biocompatibility or toxicity risks that require thorough evaluation. Finally, therapeutic limitations have been reported in stroke models, where intranasally delivered neural stem cells achieved lower and slower efficacy compared with intravenous or intracerebroventricular routes [191]. In addition, recombinant VEGFD were difficult to detect in brain lysates despite functional benefits [93]. Furthermore, only a small proportion of nasally delivered stem cells successfully entered the CNS [57].

To fully realize the potential of IN delivery for stroke therapy, future research should focus on optimizing formulations and delivery systems, deepening mechanistic insights, and expanding clinical translation. Advances in formulation technologies—including safer nanoparticle platforms, precision dosing, cytosolic delivery surface functionalization, and controlled-release systems—are required to overcome toxicity and improve therapeutic durability [78]. Targeted strategies using ligands, peptides, or optimized lipid nanoparticles (LNPs) for circular RNA delivery hold promise for enhancing brain specificity while minimizing systemic exposure [16]. Future research must investigate how nanocarriers interact with biologically abundant proteins [192]. It is also necessary to conduct real-time bioimaging experiments to reveal how these nanocarriers pass through the olfactory bulb and adjacent biological tissues [17,193]. Mechanistic studies should further elucidate how drugs and nanocarriers interact with nasal–brain pathways, with particular emphasis on the role of perivascular spaces (PVS) as conduits for bulk flow and convection. Researchers must also clarify the contributions of olfactory and trigeminal nerve routes in this process. In parallel, extensive toxicological evaluation—including chronic safety, biodegradability, and immunogenicity—must be conducted, supported by comprehensive pharmacokinetic and pharmacodynamic studies. Expanding the therapeutic window, validating efficacy across more physiologically relevant preclinical models such as non-human primates, and testing larger biologics including monoclonal antibodies will also be critical steps. Together, these research directions will help establish intranasal delivery as a safe, effective, and non-invasive therapeutic strategy that bypasses the BBB and provides targeted interventions for ischemic stroke and other CNS disorders.

Ultimately, advancing intranasal delivery strategies holds the potential to transform ischemic stroke treatment by offering a non-invasive, BBB-bypassing route that enables safer and more effective therapeutic access to the brain.

## Figures and Tables

**Table 1:** Summary of preclinical studies on intranasal therapeutics for ischemic stroke (N/A: not available).

Drug Class	Therapeutic Substance	Size/Charge	Period of Administration (Loading Dose)	Proposed Mechanism	Outcome	Model	References
Lipid derivative small molecule	Elovanoids	N/A	3 h, 24 h, 48 h after stroke (20 μg/20 μL)	Downregulation of pro-inflammatory gene; Upregulation of anti-inflammatory and pro-homeostatic genes	↓ Infarct volume ↓ Neurological deficits ↑ Sensorimotor functional recovery ↑ Angiogenesis	Male SD rats, tMCAO	[84]
Lipid derivative small molecule	Sodium Butyrate (NaB)	N/A	1 h after stroke (2.5 mg/kg, 7.5 mg/kg, 22.5 mg/kg; total 24 μL)	Reducing apoptosis via GPR41-Gβγ-PI3K-Akt pathway.	↓ Infarct volume ↓ Neurological deficits ↑ Sensorimotor functional recovery	Male SD rats, tMCAO	[85]
Synthetically derived small molecule	(+)-Naloxone (Naloxone enantiomer)	N/A	1 d after stroke, repeated (twice a day for 7 days) (10 μL)	Decreasing microglia/macrophage activation	↓ Infarct volume ↓ Neuronal loss ↑ Sensorimotor functional recovery	Male SD rats, dMCAO	[86]
Synthetically derived small molecule	Edaravone-IL (Ionic Liquid formulation)	182.4 ± 43.2 nm/−38.6 ± 4.3 mV	Immediately after stroke (20~200 µg/mL)	Antioxidation	↓ Infarct volume ↓ Neurological deficits	Male Wistar rats, tMCAO	[87]
Synthetically derived small molecule	BNPs-PAMAM/DEX (Dexmedetomidine loaded cluster)	129.4~136.2 nm/−33.61 ± 1.3 mV	30 min. after stroke (0.675 mg/mL)	Antioxidation; anti-inflammation; enhanced mitochondrial autophagy via α2-adrenoceptor activation	↓ Infarct volume ↓ Neurological deficits ↓ Neuronal apoptosis	SD rats, tMCAO	[88]
Growth Factor	AAV-BDNF, AAV-TrkB	N/A	1 d after stroke (2 × 10^10^ viral genomes in 10 × 6 μL drops)	Increasing synaptic plasticity and connectivity	↑ Sensorimotor functional recovery ↑ neurotransmission efficiency	Male SD rats, pMCAO	[89]
Growth factor	IGF-I (Insulin-like growth factor-I)	N/A	10 min, 24 h, 48 h after stroke (75 μg, 150 μg; in 50 μL)	-	↓ Infarct volume ↑ Sensorimotor functional recovery	Male SD rats, MCAO	[90,91]
Neurotrophic factor	rhMANF (Recombinant Human MANF)	N/A	12 h before, Immediately before, Immediately after stroke (20 μg or 60 μg in 10 μL)	Downregulation of pro-inflammatory cytokines; upregulation of anti-inflammatory cytokines	↓ Infarct volume ↓ Neurological deficits	Male SD rats, dMCAO	[92]
Growth Factor	VEGFD Mimetics	N/A	10 min, 24 h, 48 h after stroke (1 μg in 20 μL)	Preservation of synaptic connectivity and dendritic structure	↓ Infarct volume ↑ Sensorimotor functional recovery	Male C57 mice, MCAO	[93]
Glycoprotein	Wnt3a	N/A	1 h after stroke, once a day for 3 or 7 days (2 μg/kg in 25 μL)	Upregulation of BDNF; Promoting neurogenesis, angiogenesis	↓ Infarct volume ↑ Sensorimotor functional recovery	Male C57 mice, pMCAO	[94]
Glycoprotein	Wnt3a	N/A	1 h after stroke (2 μg/kg in 25 μL)	Neuroprotection via Frizzled-1/PIWIL1/FOXM1 pathway	↓ Infarct volume ↓ Neurological deficits ↓ Neuronal apoptosis	Male and female SD rats, tMCAO	[95]
Cytokine	IL-13	N/A	2 h after stroke, then daily for 7 days (60 μg/kg; 150 μg/mL)	Increasing anti-inflammatory macrophages via inhibition of STAT3 phosphorylation	↑ Sensorimotor functional recovery ↑ White matter integrity	Male C57 mice, tMCAO	[96]
Cytokine	IL-4 Nanoparticles	122.6 ± 2.4 nm/4.05 ± 1.35 mV	6 h after stroke, repeated for days 1–7, 14, 21, and 28 (5~100 ng/mL)	OPC differentiation via PPARγ pathway; Anti-inflammatory microglia polarization	↓ Infarct volume ↑ Sensorimotor functional recovery ↑ White matter integrity	Male C57 mice, tMCAO	[97]
Complement peptide	C3a	N/A	Daily from 7 to 21 or 28 days after stroke (200 nM in 20 μL)	Stimulation of neural plasticity; reducing astrocyte reactivity; Upregulation of Igf1, Thbs4	↑ Sensorimotor functional recovery ↑ Cortical connectivity	Male C57 mice, PT	[98,99]
Phosphoglycoprotein	Osteopontin	N/A	10 min. 1, 3, 5 h after stroke (5 mg in 50 μL)	-	↓ Infarct volume ↓ Neurological deficits	Male and female C57 mice, tMCAO	[100]
Peptide	Osteopontin peptide	183 ± 57.3 nm [101], N/A [102,103]/N/A [101,102,103]	1, 3, 6 h after stroke (1 μg [101], 500 ng [103], 10–1000 ng [102])	Anti-inflammation; Enhanced phagocytosis	↓ Infarct volume ↓ Neurological deficits	Male SD rats, tMCAO	[101,102,103]
Enzyme	tPA, tPA-S478A (protease-inactive)	N/A	7, 9, 11, 13 days after stroke or 6 h after stroke and repeated every 2 days for 14 days (300 μg [104], 2 mg/kg [105])	Sprouting and outgrowth of axons	↑ Sensorimotor functional recovery ↑ Axonal remodeling ↑ White matter integrity	Male CST-YFP mice, C57 mice, pMCAO	[104,105]
Antibody	Anti-NogoA	N/A	Daily for 14 days after stroke (0.4 mg/mL, 4.16 mg/mL, 4.167 mg/mL in total 24 μL)	Inhibition of neurite growth inhibitor (Nogo-A)	↑ Sensorimotor functional recovery ↑ Axonal remodeling	Female Long-Evans rats, PT	[81]
Gene therapy (siRNA)	SRSF3-siRNA	N/A	24 h after stroke (15 mg/mouse)	Inhibition of SRSF3, leading to restoration of immune mRNA; Increased microglia activation and TLR2 signaling	↓ Infarct volume	Male C57 mice, tMCAO	[106]
Gene therapy	Sirt1 targeting CRISPR/dCas9 system	43 nm/3.2 mV	3 h after stroke (10 μg of dCas9, 6 μg of gRNA in 100 μL)	Anti-apoptosis and neuroprotection via Sirt1 upregulation	↓ Cell death	Male BALB/c mice, pMCAO	[107]
Gene therapy (circularRNA)	circSCMH1 RNA	140~170 nm/−1.15~−3.2 mV	24 h after stroke (12, 36, 120 μg/kg)	Promotion of white matter repair; Reduction of microglia and astrocyte response	↑ Sensorimotor functional recovery ↑ Cognitive functional recovery ↑ Angiogenesis	Male C57 mice, PT	[80]
Cell therapy	Hypoxic-preconditioned BMSCs	N/A	24 h after stroke (1 × 10^6^ cells/100 μL)	Immunosuppression; upregulation of migration-related proteins (CXCR4) and MMP-2,-9	↓ Infarct volume ↓ Cell death ↑ Sensorimotor functional recovery	Male C57 mice, pMCAO	[108]
Cell therapy	BMSC + IGF-1	N/A	3, 5, 7 d after stroke (1 × 10^6^ cells/100 μL + 500 ng (IGF1))	Increased neurotrophic and angiogenic factors (BDNF, VEGF, Ang-1)	↑ Neurovascular regeneration ↑ Cerebral blood flow ↑ Sensorimotor functional recovery	Male C57 mice, pMCAO	[109]
Cell therapy	MSC-derived supernatant/cell lysate	N/A	Daily for 3 days after stroke (100 μL of supernatant or 1 mg/mL, 2 mg/mL lysate)	Promotion of anti-inflammatory neutrophil via PPAR-γ/STAT6/SOCS1 pathway	↓ Infarct volume ↑ Angiogenesis	Male C57 mice,	[110]

**Table 2:** Comparison of MSC-based therapeutic strategies.

Type	Advantages/Mechanisms	Limitations/Challenges	Clinical Translation Status
**Live MSCs**	Self-renewal and paracrine abilities; easy to isolate with few ethical concerns; promote neuroprotection, regeneration, and inflammation control. [57,58,151]	Risk of entrapment in peripheral tissues (e.g., lungs) after IV injection; potential for embolism or hemorrhage; low post-transplantation survival rate; inconsistent clinical trial results. [57,152,153]	Most extensively studied in stroke. Multiple Phase I/II trials have confirmed safety, but Phase II/III trials have shown inconsistent efficacy, often failing to meet primary endpoints. [40]
**Preconditioned MSCs**	Pre-treatment (e.g., with hypoxia or chemicals) enhances cell survival, homing, proliferation, and secretion of therapeutic factors, improving neuroprotective and migratory capabilities. [108,154,155]	Optimal preconditioning protocols (dose, timing, specific agents) are yet to be fully established. [57]	Modified BM-MSCs (SB623) have shown safety and clinical improvement in a Phase 1/2a study for chronic stroke. [156]
**MSC + Bioactive Factors (e.g., IGF-1)**	Genetic engineering to overexpress specific neurotrophic factors (e.g., BDNF, VEGF, IGF-1) significantly enhances the neuroprotective and regenerative effects of the cells. [109,157]	Primarily explored as a preclinical strategy to boost therapeutic efficacy. [57]	Mostly in the preclinical stage of research [40]
**MSC-derived EVs**	The cell-free approach avoids risks of live cell therapy. EVs contain therapeutic cargo (proteins, RNA) that can reduce apoptosis, promote angiogenesis, and exert immunomodulatory and neuroprotective effects comparable to live cells. [158,159,160,161]	Mechanisms of action require further clarification; optimization of isolation and production is needed. [40]	A promising future direction for enhancing efficacy, but currently concentrated in the preclinical research phase. [40,58]

**Table 3:** Comparison of different nanocarrier types for nose-to-brain delivery in stroke therapy.

Nanoparticle Type	Advantages for Brain Penetration and Efficacy	Limitations and Translational Hurdles
Extracellular Vesicles (EVs)	- Endogenous Origin - Offering high biocompatibility and low immunogenicity. - Possess natural targeting capabilities.	- Prone to physical (mucociliary) clearance and enzymatic degradation, leading to instability and rapid disintegration. - Insufficient quantities from autologous cells, creating a major large-scale production bottleneck. - Use of allogenic cells introducing immune response risks.
Liposomes	- High structural stability and longer circulation times compared to EVs. - Tunable for high drug-loading capacity. - Manufacturing is easier to scale up compared to EVs.	- Synthetic nature may lead to lower biocompatibility or higher immunogenicity compared to EVs. - Precise transport mechanisms remain unclear for many formulations. - Non-specific delivery must be carefully assessed.
Polymer-based carriers (e.g., Gelatin, Chitosan, Poloxamer)	- Excellent mucoadhesive properties (e.g., chitosan, polyacrylates) enhance nasal residence time by resisting mucociliary clearance. - Can be designed for sustained drug release or sol–gel phase transitions, which also prolong contact time. - Can enhance absorption and target specific sites.	- Biocompatibility and potential long-term toxicity of synthetic polymers require thorough evaluation. - Size/composition distribution per batch must be fully assessed and optimized. - Type of drug and drug-loading capacity is strictly limited by the structure of polymer.
Inorganic Nanoparticles (e.g., Gold, CaP, Mn/Co_3_O_4_)	- Highly tunable surfaces for conjugating targeting ligands (e.g., ApoE-mimetic peptides) - Some types (e.g., CaP) offer high biocompatibility and biodegradability - Intrinsic therapeutic properties (e.g., ROS scavenging) - Enable theranostic applications (e.g., NIR imaging and drug delivery)	- Precise delivery pathways from the nasal cavity to the brain remain to be fully elucidated for many inorganic NPs. - Long-term toxicity, accumulation, and biodegradability must be carefully assessed.

## Data Availability

Not applicable.

## Abbreviations

AAV	Adeno-Associated Virus
ABC	Arachnoid Barrier Cell
ADP	Adenosine diphosphate
Akt	A serine/threonine-specific protein kinase
AMP	Adenosine monophosphate
ATP	Adenosine triphosphate
BBB	Blood–Brain Barrier
BDNF	Brain-Derived Neurotrophic Factor
BMSC	Bone Marrow–Derived Mesenchymal Stem Cell
BMSC-EV	Bone Marrow–Derived Mesenchymal Stem Cell Extracellular Vesicle
BNP	Bioadhesive Nanoparticle
bFGF	Basic Fibroblast Growth Factor
bPEI	Branched Polyethylenimine
CaPNP	Calcium Phosphate Nanoparticle
CD	Cyclodextrin
circRNAs	Circular RNAs
CNS	Central Nervous System
CRISPR	Clustered Regularly Interspaced Short Palindromic Repeats
CSF	Cerebrospinal Fluid
DEX	Dexmedetomidine
DFO	Deferoxamine
dMCAO	Distal Middle Cerebral Artery Occlusion
DSPE	1,2-Distearoyl-sn-glycero-3-phosphoethanolamine
EDA	Edaravone
EGFR	Epidermal Growth Factor Receptor
ELVs	Elovanoids
EV	Extracellular Vesicle
FOXM1	Forkhead box M1
Frz1	Frizzled-1
GDF10	Growth Differentiation Factor10
GNP	Gold Nanoparticle
GPR41	G Protein-coupled Receptor 41
GTP	Guanosine triphosphate
HA	Hyaluronic Acid
HMOX1	Heme Oxygenase 1
HP-BMSC	Hypoxia-Preconditioned Bone Marrow–Derived Mesenchymal Stem Cell
IA	Intra-arterial
IC	Intracerebral
ICV	Intracerebroventricular
IGF-1	Insulin-like Growth Factor1
IL-10	Interleukin-10
IL-13	Interleukin-13
IL-1β	Interleukin-1β
IL-4	Interleukin-4
IL-6	Interleukin-6
ILs	Ionic Liquids
IN	Intranasal
iNOS	Inducible nitric oxide synthase
i.p.	Intraperitoneal
IT	Intrathecal
IV, i.v.	Intravenous
JNK	c-Jun N-terminal kinase
LILRB4a	Leukocyte Immunoglobulin-Like Receptor subfamily B member 4a
LNP	Lipid Nanoparticle
MANF	Mesencephalic Astrocyte-Derived Neurotrophic Factor
MAPK	Mitogen-activated protein kinase
MCAO	Middle Cerebral Artery Occlusion
MDM	Monocyte-Derived Macrophage
MMP	Matrix Metalloproteinase
MPTP	Mitochondrial permeability transition pore
MSC	Mesenchymal Stem Cell
Muse cell	Multilineage-Differentiating Stress-Enduring Cell
N2B	Nose-to-brain
NALT	Nasopharynx-associated lymphoid tissue
NBT	Nanoparticle-Based Therapeutic
NET	Neutrophil Extracellular Trap
NF-κB	Nuclear Factor Kappa B
NHPs	Non-human primates
NIR	Near-Infrared
NK	Natural killer
NMDA	N-Methyl-D-Aspartate
NMDAR	N-Methyl-D-Aspartate Receptor
Nogo-A	Neurite Outgrowth Inhibitor-A
NOX	NADPH Oxidase
NP	Nanoparticle
NPC	Neural Progenitor Cell
NSC	Neural Stem Cell
NT	Nucleotide
NTR	Normotensive rats
OECs	Olfactory ensheathing cells
ONF	Olfactory Nerve Fibroblast
OPC	Oligodendrocyte Progenitor Cell
OPN	Osteopontin
OSN	Olfactory Sensory Neuron
PAIS	Perinatal Arterial Ischemic Stroke
PAMAM	Polyamidoamine
PARP-1	Poly(ADP-ribose) Polymerase1
PASSIoN trial	Perinatal Arterial Stroke treated with Stromal cells IntraNasally trial
PEG	Polyethylene Glycol
PEI	Polyethylenimine
PI3K	Phosphatidylinositol 3-kinase
PIWI1a	PIWI-like protein 1a
PLA-HPG	Polylactic acid-hyperbranched polyglycerol
pMCAO	Permanent Middle Cerebral Artery Occlusion
PNS	Panax Notoginseng Saponins
PPARγ	Peroxisome Proliferator-Activated Receptor Gamma
PT	Photothrombosis
PU	Puerarin
PVS	Perivascular Space
Rg3	Ginsenoside Rg3
ROS	Reactive Oxygen Species
SAS	Subarachnoid space
s.c.	Subcutaneous
SCMH1	Scm Polycomb Group Protein Homolog 1
SD	Sprague Dawley
sEV	Small Extracellular Vesicle
SGRNA	Single-Guide RNA
SHp	Stroke-Homing Peptide
SIRT1	Silent Information Regulator1
SSEA-3	Stage-specific embryonic antigen-3
ST2	Suppressor of Tumorigenicity
STAT3	Signal Transducer and Activator of Transcription 3
STAT6	Signal Transducer and Activator of Transcription 6
TIMP1	Tissue Inhibitor of Metalloproteinases 1
TJs	Tight junctions
TK	Thioketal
TLR4	Toll-like Receptor 4
tMCAO	Transient Middle Cerebral Artery Occlusion
TNF-α	Tumor Necrosis Factor α
tPA	Tissue Plasminogen Activator
VEGF	Vascular Endothelial Growth Factor
VEGFD	Vascular Endothelial Growth Factor D
VLC-PUFAs	Very long-chain polyunsaturated fatty acids
Wnt3a	Wingless-Type MMTV Integration Site Family, Member 3A
